# Surface Electromyography for Parkinson’s Disease Monitoring: A Review of Machine and Deep Learning Techniques

**DOI:** 10.3390/s26102927

**Published:** 2026-05-07

**Authors:** Sara Bruschi, Marco Esposito, Sara Raggiunto, Luisiana Sabbatini, Alberto Belli, Michele Paniccia, Paola Pierleoni

**Affiliations:** 1 Department of Information Engineering (DII), Università Politecnica Delle Marche, 60131 Ancona, Italy; m.esposito@univpm.it (M.E.); s.raggiunto@univpm.it (S.R.); l.sabbatini@univpm.it (L.S.); p.pierleoni@univpm.it (P.P.); 2 Rehabilitation Center S. Stefano, 62018 Potenza Picena, Italy

**Keywords:** surface electromyography, Parkinson’s disease, wearable sensors, machine learning, deep learning, tremor analysis, gait analysis, freezing of gait, biomedical signal processing

## Abstract

Parkinson’s disease (PD) is a neurodegenerative disorder affecting millions worldwide, characterized by motor symptoms such as tremor, rigidity, and bradykinesia that significantly impair daily life. The current diagnosis and monitoring rely primarily on clinical observations and rating scales (e.g., the MDS-UPDRS), which are subjective and limited in detecting subtle motor alterations, leading to inter- and intra-rater variability. In recent years, wearable sensors such as surface electromyography (sEMG) and inertial measurement units (IMUs) have emerged as non-invasive tools for quantifying neuromuscular activity and motor performance in PD. When combined with machine learning (ML) and deep learning (DL) techniques, these signals enable the development of models for disease detection, patient classification, and symptom severity assessment. This review provides a structured overview of recent ML and DL approaches applied to surface electromyography for PD monitoring, addressing a gap in the current literature. It analyzes data acquisition strategies, preprocessing techniques, feature extraction methods, model architectures, and evaluation protocols across tasks such as diagnosis, tremor analysis, freezing of gait detection, and gait assessment. Despite promising results, key challenges remain, including limited dataset size, lack of standardization, and poor generalization. Finally, this work highlights emerging trends and identifies a representative processing pipeline to support real-world clinical translation.

## 1. Introduction

Parkinson’s disease (PD) is one of the most common neurodegenerative movement disorders [1], affecting millions of people worldwide and projected to impact approximately 9 million individuals over the age of 50 by 2030 [2]. It is primarily characterized by the progressive loss of dopaminergic neurons in the substantia nigra pars compacta, leading to a wide range of motor and non-motor symptoms [3]. Motor manifestations, including tremor, rigidity, bradykinesia (slow body movements), freezing of gait, and dyskinesia (involuntary muscle movements), significantly impair patients’ daily functioning, while non-motor symptoms, such as cognitive decline, fatigue, and depression, further contribute to disease burden [4]. Although several genetic and environmental factors (such as family history and genetics, smoking, caffeine, exposure to chemicals and pesticides) have been associated with PD, its etiology still remains unclear in most cases [5,6]. There is currently no cure for Parkinson’s disease as the available therapies cannot arrest or reverse the disease’s progression. The current treatments, such as the gold standard Levodopa administration, provide symptomatic relief but do not halt disease progression [7]. Consequently, there is a growing need in the research field for objective and reliable tools for diagnosis, assessment and monitoring of the disease.

The diagnosis of PD is primarily clinical and relies on the identification of cardinal motor symptoms, including resting tremor, rigidity, and bradykinesia, as well as the patient’s response to dopaminergic therapy [8]. Neuroimaging techniques, such as CT, MRI, and DaTscan, may support differential diagnosis by excluding other conditions (e.g., brain tumor or stroke) or assessing dopaminergic deficits [9]. In clinical practice, neurologists rely on clinical scales for the evaluation of the severity of motor symptoms [10]. The Movement Disorder Society-sponsored revision of the Unified Parkinson’s Disease Rating Scale (MDS-UPDRS) is the most popular rating scale used for the assessment of PD motor impairments. It assigns a score of 0 to 4 according to the severity of the symptom under evaluation [11]. The Hoehn and Yahr scale (H&Y) includes five stages (from 1 to 5) and is used to assign an overall score to the patient based on disease progression [12]. These scoring systems are inherently subjective, depending on clinician expertise and judgment, and are affected by inter- and intra-rater variability.

In this context, the development of wearable sensor technologies has enabled the acquisition of objective and quantitative data for PD assessment. The data obtained from these systems support applications such as early diagnosis, disease classification, differential diagnosis, and continuous monitoring of motor symptoms [13]. Currently, among the most widely used devices in the evaluation of PD and movement disorders are inertial measurement units (IMUs), which include accelerometers, gyroscopes, and magnetometers [14]. Increasing attention has also been given to electromyographic (EMG) signals, particularly surface electromyography (sEMG), which provides direct information on muscle activation patterns. sEMG signals have been shown to capture characteristic features associated with tremor, postural instability, and bradykinesia, offering valuable insights into neuromuscular alterations in PD [15].

In recent decades, artificial intelligence (AI), and in particular machine learning (ML) and deep learning (DL) techniques, have been increasingly applied to the analysis of biomedical signals for the diagnosis and monitoring of movement disorders, including Parkinson’s disease [16]. These approaches enable the extraction of complex patterns from high-dimensional data, supporting objective and automated classification, prediction, and quantitative assessment of disease-related features. Furthermore, the integration of signal-based biomarkers with clinical and physiological data has the potential to improve early detection and personalized disease management [17].

The application of ML and DL techniques to sEMG signals in PD has recently gained increasing attention. Several studies have demonstrated that sEMG signals capture characteristic neuromuscular patterns associated with PD, enabling the discrimination between patients and healthy subjects, as well as the assessment of symptom severity and the detection of specific motor impairments. In addition, sEMG is often combined with other sensing modalities, particularly inertial measurement units, with multimodal approaches consistently showing improved performance compared to unimodal configurations. These developments highlight the potential of ML- and DL-based sEMG systems as objective and quantitative tools to support clinicians’ decision-making, reducing the subjectivity associated with traditional rating scales, such as the MDS-UPDRS and H&Y.

Despite these promising results, the existing literature remains highly heterogeneous, with significant variability in datasets, acquisition protocols, preprocessing pipelines, feature extraction strategies, and model selection criteria. Moreover, a structured and task-oriented analysis of studies focusing on ML and DL approaches applied to sEMG data in Parkinson’s disease is still lacking in the literature. To address this gap, this structured narrative review provides a systematic overview of the recent studies published over the last decade, examining the methodological trends and performance across different clinical tasks, including disease diagnosis, symptom assessment, and progression monitoring.

### Contributions

This review provides a comprehensive and structured analysis of recent studies applying artificial intelligence techniques to surface electromyography (sEMG) signals in Parkinson’s disease. The main contributions are summarized as follows:

- A systematic identification and analysis of the recent literature on the application of machine learning (ML) and deep learning (DL) models to sEMG data in Parkinson’s disease;
- A comparative evaluation of data acquisition strategies across different clinical tasks, highlighting the variability in experimental setups and protocols;
- A detailed analysis of signal preprocessing techniques (e.g., filtering and normalization) and their impact on model performance;
- A critical review of feature extraction approaches, identifying the most informative and commonly adopted features for PD-related tasks;
- A comparative assessment of ML and DL models, discussing their performance, advantages, and limitations in different application scenarios;
- The identification of a representative processing pipeline, along with a discussion of the current limitations and future research directions toward clinically applicable solutions.

The remainder of the paper is organized as follows. Section 2 introduces the background concepts, including Parkinson’s disease (Section 2.1), wearable sensing technologies (Section 2.2), surface electromyography (Section 2.3), and ML and DL methodologies for biomedical signal analysis (Section 2.4). Section 3 describes the review methodology and outlines the research questions (Section 3.2). Section 4 presents the main analysis of the selected studies, organized by clinical task, including diagnosis and severity assessment (Section 4.1), differential diagnosis (Section 4.2), freezing of gait detection (Section 4.3), tremor analysis (Section 4.4), and gait and other motor symptoms (Section 4.5). Section 5 provides a critical discussion of the findings, focusing on datasets, methodologies, current limitations and real-world implementations. Finally, Section 6 concludes the paper and outlines future research directions.

## 2. Background

This section provides the fundamental background necessary to contextualize this review. Specifically, it introduces key concepts related to Parkinson’s disease, wearable sensing technologies, surface electromyography (sEMG) signals, and ML/DL-based methodologies for biomedical signal analysis, which form the basis of the studies discussed in this work.

### 2.1. Parkinson’s Disease

Parkinson’s disease (PD), first described by James Parkinson in his *Essay on the Shaking Palsy* [18], is a neurodegenerative disorder characterized by the accumulation of Lewy bodies containing α-synuclein in the substantia nigra and the progressive loss of dopaminergic neurons in the pars compacta. This degeneration leads to motor impairments that significantly affect the quality of life of patients [6]. Symptoms typically emerge when a substantial proportion of these neurons is lost [19].

The annual incidence of PD varies between fewer than 10 and more than 20 cases per 100,000 individuals, depending on demographic and diagnostic factors, with under-diagnosis being particularly common [6]. Establishing a correct diagnosis process is critical; however, it remains challenging and is primarily based on clinical criteria based on the identification of cardinal motor symptoms. Although standardized frameworks, such as the UK Brain Bank Criteria, have improved diagnostic accuracy, misdiagnosis remains common, particularly in early stages [20]. To address this, the Movement Disorder Society (MDS) introduced revised diagnostic criteria to improve reproducibility and applicability. This framework maintains rigidity, rest tremor, and bradykinesia as cardinal features of the disease and incorporates exclusion criteria (to rule out PD), red flags (requiring additional supporting evidence) and supportive criteria (positive features that favor the diagnosis) [21]. Validation studies on 434 PD patients and 192 non-PD patients confirmed that the revised MDS criteria achieved higher sensitivity and specificity than the Brain Bank in PD diagnosis [22].

Clinically, PD is characterized by both motor and non-motor symptoms, some of which can be provoked or aggravated by dopaminergic treatments [23]. The main motor features are summarized by the acronym TRAP—tremor at rest, rigidity, akinesia (bradykinesia) and postural instability—and are central to diagnosis. Additional impairments, such as reduced arm swing, freezing of gait (FoG), and balance difficulties, further increase the risk of falls and negatively impact quality of life [24]. PD also manifests a broad spectrum of non-motor symptoms that typically worsen with illness progression. These include mood disorders, apathy, depression, cognitive dysfunctions, sleep disorders, irritability, and hallucinations [25]. Screening non-motor signs, as well as motor cardinal features, might help with a preclinical diagnosis [26]. Based on these symptoms, a number of rating scales are used for the evaluation of motor impairment [24]: the H&Y scale is used to compare groups of patients and to perform a general evaluation of disease progression with a score of 0 (no sign) to 5 (wheel-chaired or bedridden) [12]; the UPDRS is the most prevalent scale to track PD aggravation through a severity point system assigned to each symptom [11].

A major limitation of current clinical practice is the subjectivity of diagnostic and assessment procedures. Diagnosis is based primarily on observation of motor and non-motor symptoms, requiring significant clinical expertise and potentially leading to variability between clinicians. The quantification of severity using rating scales is influenced by both patient conditions and the experience of the evaluator. Assessments are typically performed in controlled clinical settings, which may not accurately reflect real-world motor behavior of patients. As a result, both diagnosis and monitoring are affected by inter- and intra-rater variability [14].

These limitations highlight the need for objective quantitative biomarkers that are capable of capturing subtle motor alterations, which motivates the use of sensor-based approaches. Consequently, recent research has increasingly focused on developing techniques that provide objective and quantifiable measurements to support clinical decision-making. Such tools have the potential to improve diagnostic reliability and enable more consistent and accurate monitoring of disease progression.

### 2.2. Wearable Sensors for Parkinson’s Disease

The current healthcare system is shifting toward a new era of diagnostic precision and personalized treatment. As the global population ages and disorders such as PD become more prevalent, there is a growing need for reliable cost-effective tools for disease diagnosis and assessment [27]. In clinical practice, PD severity is typically evaluated using standardized rating scales, which remain subjective and dependent on clinician expertise. Moreover, symptoms can fluctuate over time, and discrepancies between clinical evaluations and patient-reported outcomes are common and complicate treatment decisions [28,29]. These limitations highlight the need for objective and continuous monitoring tools [14] to improve patient–clinician communication, enable timely therapeutic adjustments, and enhance disease management [29]. In this context, wearable technologies and the Internet of Things (IoT) enable non-invasive and continuous measurement of physiological and kinematic data, supporting improved disease management [27].

Wearable sensors such as smartwatches, inertial units, or electromyography bands, worn on the body or integrated into clothing to record physiological and kinematic data [14], allow monitoring outside controlled clinical environments, facilitating early diagnosis, differential diagnosis, and quantitative symptom assessment [30]. Among these, inertial measurement units (IMUs) (accelerometers, gyroscopes, and magnetometers), paired with wireless communication technologies to ensure low power consumption, comfort, and ease of use, are widely used for gait and tremor analysis, with applications spanning diagnosis, motor assessment, and long-term monitoring of disease progression [13].

Several studies have demonstrated the effectiveness of wearable systems for PD monitoring. Lee et al. [31] developed a dual-sensor system worn on the feet and waist, integrating plantar pressure sensors and postural sway modules with triaxial accelerometers and gyroscopes. They provide quantitative gait statistics to differentiate normal from PD walking patterns. Pierleoni et al. [32] proposed a single wireless IMU placed on the lower back for continuous patient monitoring. This compact and non-invasive system achieved an absolute percentage error of approximately 2% in estimating gait-related parameters, such as step count, under clinical conditions and showed strong agreement with video recordings and diary evaluations in home settings. This confirms the reliability of wearable-based monitoring. San-Segundo et al. [33] demonstrated the feasibility of a wrist-worn accelerometer for real-world PD tremor assessment. Even without video annotations, their model effectively replicated patient diaries, confirming that a small, low-cost, and non-invasive sensor can support reliable symptom quantification.

More recently, multimodal approaches combining IMU, EMG, and other biosignals (ECG or EEG) have been explored to enhance the accuracy of PD symptom recognition. Hybrid systems provide complementary information on muscular and kinematic activity, improving robustness and diagnostic accuracy. In particular, electromyography (EMG) provides direct insight into muscle activation patterns associated with PD. Studies have shown that PD patients exhibit alternating contractions of antagonistic muscles, while essential tremor (ET) is characterized by synchronous activation, suggesting their potential as differential biomarkers [34]. However, conventional EMG systems are expensive, intrusive, and require skilled operators, limiting their applicability. Vescio et al. [35] developed and validated a low-cost wearable device, *μ*EMG, for quantitative characterization of phase displacement between antagonistic muscles. Their device achieved performance comparable to standard EMG, accurately distinguishing PD from ET subjects in clinical settings. Surface electromyography (sEMG) has emerged as a non-invasive alternative, enabling the acquisition of muscle electrical activity through skin-mounted electrodes rather than intramuscular needles. This approach supports continuous, comfortable, and real-world monitoring of symptoms, facilitating the extraction of quantitative biomarkers for PD assessment.

Overall, wearable technologies enable objective monitoring of motor symptoms in real-world settings, overcoming the limitations of episodic clinical evaluations. sEMG is particularly promising due to its ability to directly capture muscle activation patterns and provide insight into the neuromuscular mechanisms underlying motor impairments. Its non-invasive nature and versatility make it well suited for the assessment of key symptoms, such as tremor, rigidity, and bradykinesia.

Building on these considerations, the following sections focus on machine learning and deep learning approaches applied to sEMG signals for Parkinson’s disease analysis, with emphasis on data acquisition, diagnostic applications, symptom quantification, and disease monitoring.

### 2.3. Surface Electromyography

Electromyography (EMG) measures muscle activity by recording the electrical signals generated during muscle contraction, reflecting neuromuscular activation processes. Myoelectric signals occur from changes in the potential of the muscle fiber membrane during voluntary or involuntary contractions [36] and can be obtained “directly” (intramuscular needles) or “indirectly” with surface electrodes placed on the skin. Due to its non-invasiveness, surface EMG (sEMG) is widely used in research and clinical settings [37,38] to assess muscle activation, coordination, fatigue and neuromuscular control [39]. The sEMG signal reflects the timing and intensity of neuromuscular activation and originates from the depolarization and repolarization of muscle fiber membranes during contraction, which correspond to the superposition of motor unit action potentials (MUAPs), detected on skin surface and generated by active motor units (MUs), each consisting of a motoneuron and its innervated muscle fibers [37]. As a result, sEMG is a complex and stochastic signal [36] typically characterized by low amplitudes (tens of μV to a few mV) and a frequency range of approximately 10 to 400 Hz, with dominant energy in the 50–150 Hz band [40].

**Figure 1:**
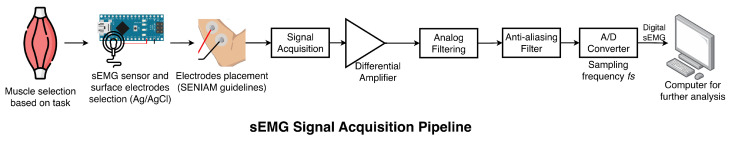
Overview of the sEMG signal acquisition pipeline, including electrode-based detection, differential amplification, analog filtering (bandpass and notch), anti-aliasing, and analog-to-digital conversion for subsequent digital processing.

Signal acquisition involves electrode-based detection, amplification, filtering, and analog-to-digital conversion, as shown in Figure 1 [41,42].

Surface electrodes can be wet (metal–skin contact with a gel), dry (direct metal–skin contact), or capacitive (no direct electrical contact) [43], with silver/silver chloride (Ag/AgCl) electrodes commonly preferred due to their stability and reduced sensitivity to motion artifacts [37]. Signal quality depends on electrode placement and configuration typically standardized according to guidelines such as SENIAM (Surface EMG for Non-Invasive Assessment of Muscles) to ensure reproducibility. The recommended placement is along the muscle belly and an interelectrode distance (IED) of about 20 mm, with 10 mm electrode diameter along with a reference electrode placed over a bony prominence [44].

sEMG recordings are affected by noise sources, including motion artifacts, electromagnetic interference, and physiological variability, which can be mitigated through preprocessing techniques such as filtering and normalization (e.g., low-frequency motion artifacts around 0–20 Hz can be attenuated with high-pass filtering; biosignal artifacts can be mitigated using a 30 Hz cut-off frequency) Proper preprocessing enables the extraction of meaningful features in the time, frequency, and time–frequency domains [39], including amplitude-based descriptors (e.g., root mean square and mean absolute value), temporal features (e.g., zero crossings), and spectral measures (e.g., PSD, mean frequency, and median frequency).

As a non-invasive and quantitative measure of muscle activity, sEMG has become increasingly relevant for the assessment of movement disorders [40]. In Parkinson’s disease, where clinical evaluation relies largely on subjective scales, sEMG provides objective and independent biomarkers of neuromuscular alterations. Compared to kinematic sensors, it provides a direct measurement of muscle activation, enabling a detailed characterization of neuromuscular dysfunction in PD. Particularly, sEMG captures electromechanical activation patterns generated by motor unit (MU) activity, reflecting both central motor control mechanisms and molecular interactions of muscle fiber cells. Movement relies on MU recruitment and firing patterns and on the balance between agonist and antagonist muscles through reciprocal inhibition. In PD, dysfunction of the basal ganglia–thalamo–cortical circuits alters these mechanisms, leading to abnormal muscle activation patterns. Clinically, these manifest as distinct motor phenotypes, including bradykinesia, hypokinesia, rigidity (hypertonia), postural instability, gait disturbances (e.g., camptocormia), and resting tremor [45]. sEMG quantitatively assesses these alterations by analyzing activation patterns across muscle groups (e.g., agonist–antagonist and flexor–extensor pairs). In particular, it evaluates (i) the relative contribution of synchronous (isotonic) versus asynchronous (isometric) contractions and (ii) the prevalence of co-contraction (simultaneous activation of antagonist muscles) versus reciprocal inhibition (alternating activation). In PD, increased co-contraction and reduced reciprocal inhibition are commonly observed, contributing to rigidity and impaired movement. Altered MU synchronization and reduced variability in firing patterns have also been associated with abnormal basal ganglia output [46]. Tremor is characterized by rhythmic bursting patterns with alternating activation of agonist and antagonist muscles, reflecting pathological oscillation within the basal ganglia–thalamo–cortical circuits [47]. Additionally, reduced sEMG amplitude and delayed muscle activation during movement and gait tasks reflect decreased central motor drive and are directly linked to bradykinesia [48]. Overall, these sEMG-derived features provide a direct link between measurable muscle activity, underlying neurophysiological dysfunction, and clinical manifestations of PD, supporting their potential role as objective biomarkers.

Several studies have demonstrated differences between PD patients and healthy controls (HCs) using sEMG. Meigal et al. (2009) [49] introduced non-linear sEMG parameters to differentiate PD patients from HC. To quantify the signal complexity, the percentage of recurrences (%REC) reflects the amount of repeating patterns in the signal, and the percentage of determinism (%DET) quantifies the proportion of structured and predictable muscle activation patterns over time. Sample entropy (SampEn) measures the irregularity and unpredictability of the signal, whereas correlation dimension (CD) estimates its dynamic complexity. Furthermore, they evaluated traditional features, such as RMS and MDF, from biceps brachii (BB) recordings. They found that non-linear parameters are better at distinguishing PD patients from controls and correlate well with UPDRS scores. In a follow-up study, Meigal et al. (2013) [50] examined the non-linear characteristics of sEMG and tremor acceleration, motivated by reports of irregular MU discharge patterns in PD, with alternating inter-spike intervals. Their findings show that sEMG signals in PD are less complex and more predictable, with higher %DET and %REC values and lower SampEn and CD, suggesting that non-linear descriptors are useful for preclinical diagnosis. Similarly, Rissanen et al. [51] combined sEMG and accelerometer during static and dynamic tasks of the BB muscles, demonstrating a clear separation between PD and HC. More recently, Pacini Panebianco et al. [52] analyzed muscle activation patterns during gait using a statistical approach and integrating measures of symmetry and co-activation. They found distinct activation timings in both gastrocnemius medialis (GM) and tibialis anterioris (TA), with delayed or altered activation regions in PD patients compared to HC. They also highlighted TA’s absence or delayed activity at the beginning of each gait cycle, which generally has a shorter and anticipated activation during the swing phase in PD.

In recent years, the integration of machine learning (ML) and deep learning (DL) techniques has shifted sEMG analysis toward data-driven approaches capable of automatically extracting discriminative patterns. These methods have demonstrated strong potential for PD detection and characterization of disease-specific neuromuscular alterations.

### 2.4. Artificial Intelligence for PD Analysis

Artificial intelligence (AI), particularly machine learning (ML) and deep learning (DL) techniques, has been increasingly applied to biomedical signal analysis for disease detection, classification, and monitoring. Unlike rule-based approaches, these methods are data-driven and can learn complex patterns directly from data, enabling more automated and generalizable diagnostic systems [53,54].

In Parkinson’s disease, ML and DL techniques are commonly applied to signals acquired from wearable sensors, such as inertial measurement units and surface electromyography. A typical analysis pipeline includes data acquisition, preprocessing, feature extraction, and model training. Traditional ML relies on handcrafted features derived from time, frequency, or time–frequency domains, whereas DL enables end-to-end learning by automatically extracting hierarchical representations from raw or minimally processed data. These techniques are particularly suitable for sEMG analysis as such signals are high-dimensional, non-linear, and variable across subjects and conditions. ML and DL models could capture subtle patterns in neuromuscular activity that may not be easily identifiable using conventional analysis methods, supporting tasks such as diagnosis, symptom quantification, and disease monitoring.

Common ML models in Parkinson’s research include support vector machines (SVMs), ensemble methods, k-nearest neighbors (kNNs), regression models, decision trees (DTs), and artificial neural networks (ANNs) [55]. DL approaches are based on deep neural networks (DNNs), which consist of two or more hidden layers, capable of automatically learning hierarchical and task-specific data representations [56]. Common architectures in biomedical applications, including PD monitoring, are fully connected neural networks (FCNNs), in which each neuron is connected to all the neurons in the subsequent layer, enabling global feature interactions; convolutional neural networks (CNNs), which use learnable convolutional filters and local receptive fields combined with pooling operations to capture spatial or temporal patterns and progressively extract higher-level features [57]; and recurrent neural networks (RNNs), which are designed to model sequential data through internal memory states and recurrent connections that capture temporal dependencies [58]. CNNs have been shown to be effective in extracting local patterns from time-series data and are generally less prone to overfitting [58,59], while RNN-based models, such as long short-term memory (LSTM) networks, are designed to capture long-term temporal dependencies through gated mechanisms that regulate information flow [60].

The following sections present the methodology adopted for this review and provide a detailed analysis of recent studies applying machine learning and deep learning approaches to sEMG signals for Parkinson’s disease assessment.

## 3. Methodology

This section outlines the methodology adopted for this review, including the literature search strategy, study selection criteria, and thematic organization of the selected works. It also defines the research questions (RQs) that guide the analysis, ensuring a structured and systematic evaluation of the existing literature. This work is intended as a structured narrative review. While a systematic search strategy with defined inclusion and exclusion criteria was adopted to ensure transparency and reproducibility, the objective was not to provide exhaustive coverage of all the available literature but rather to identify the most relevant studies in the field of sEMG-based PD assessment.

### 3.1. Methods

A structured literature search was conducted between November 2025 and January 2026 using publicly accessible databases and search engines, including IEEE Xplore and Google Scholar, which were selected to cover both engineering-oriented and interdisciplinary research. Given the relatively limited number of studies in this field, this approach was considered sufficient to capture the most relevant contributions. Therefore, the aim was to identify original research articles investigating the use of non-invasive surface electromyography (sEMG) signals in combination with machine learning and deep learning techniques for Parkinson’s disease analysis, detection, and assessment. The search was performed using combinations of keywords related to Parkinson’s disease, sEMG and artificial intelligence. Specifically, the following queries were applied to titles and abstracts: (“Parkinson” OR “PD”) AND (“EMG” OR “sEMG” OR “surface EMG” OR “electromyography” OR “non-invasive EMG”) AND (“ML” OR “machine learning” OR “DL” OR “deep learning” OR “AI” OR “artificial intelligence”).

An initial screening identified 59 potentially relevant studies. Only original full-text articles written in English and published between 2018 and 2025 were considered. Some inclusion and exclusion criteria were defined to better select manuscripts in a secondary in-depth analysis. Studies were excluded if they:

- Were not original research articles (e.g., reviews, abstracts or book chapters);
- Were not written in English;
- Were not available in full text;
- Did not involve Parkinson’s disease patients;
- Used invasive (needle) EMG instead of surface EMG;
- Did not apply machine learning or deep learning techniques;
- Did not provide sufficient methodological or performance evaluation details.

On the other hand, papers were considered eligible for the review process if they:

- Studied the classification of PD from healthy control (HC) subjects;
- Evaluated the progression of the disease through the observation of its main symptoms (e.g., gait disorders, bradykinesia, tremor, FoG, etc.);
- Analyzed the differential diagnosis of PD and other similar movement disorders.

**Figure 2:**
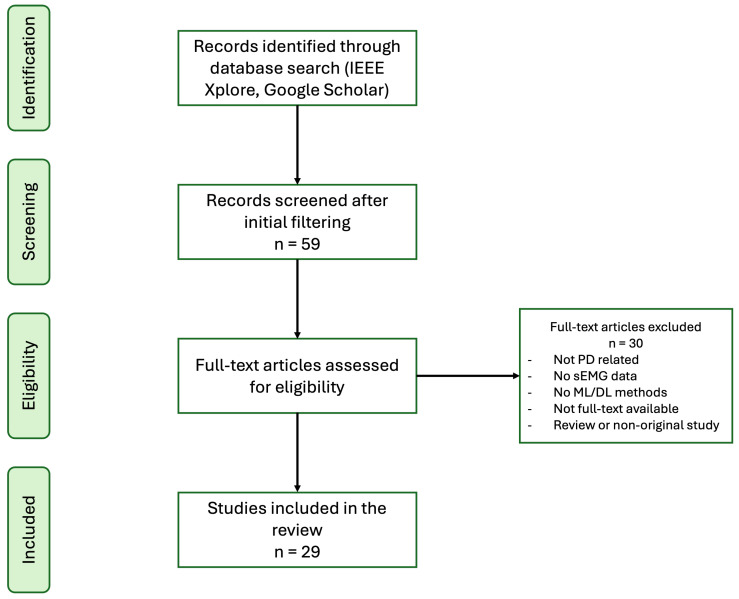
Study selection process illustrating the identification, screening, eligibility assessment, and inclusion of studies considered in this review.

After removing duplicates and applying the inclusion and exclusion criteria through title/abstract screening followed by full-text assessment, 29 studies were retained for the final review. The study selection process is summarized in Figure 2, which illustrates the identification, screening, eligibility assessment, and inclusion stages.

**Figure 3:**
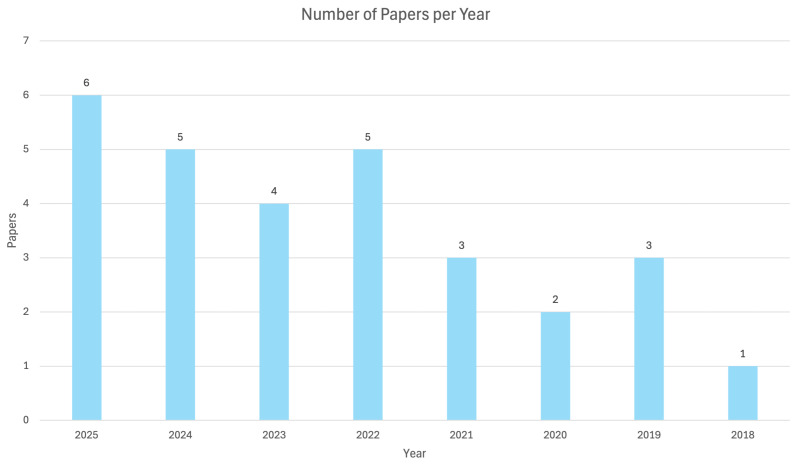
Distribution of the identified published papers per year that met the inclusion criteria of this review.

In Figure 3 the distribution of the selected articles is shown along the observation period. In recent years this topic has been increasingly addressed, and potential growth can be expected during the next few years. Furthermore, from 2018 to 2021, there were fewer relevant manuscripts that highlighted that the topic was still growing. This is also the reason why the authors decided not to go over 2018 for the review: relevant papers were not available and were not of interest for the present work.

**Figure 4:**
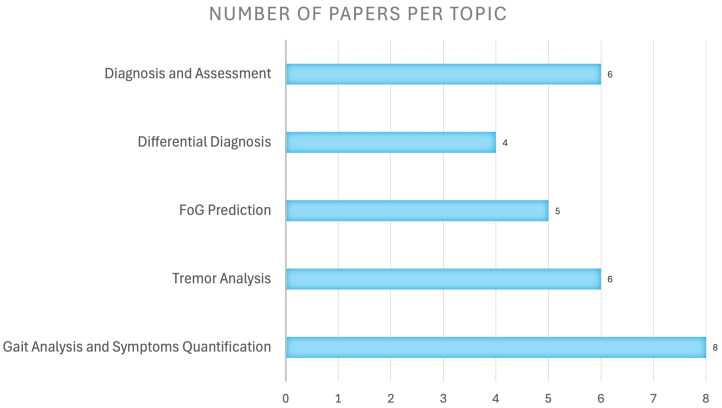
Distribution of the identified papers on ML- and DL-based analysis of the sEMG signal across the different tasks performed in relation to the analysis of Parkinson’s disease.

The selected studies were categorized based on their primary application task, including diagnosis and severity assessment of the disease (6 studies [61,62,63,64,65,66]); differential diagnosis between PD and other similar movement disorders (4 studies [67,68,69,70]); freezing of gait detection or early prediction to prevent the risk of falling (5 studies [71,72,73,74,75]); tremor analysis to detect the presence of tremor or quantify its severity (6 studies [76,77,78,79,80,81]); and gait analysis and other motor symptom quantification (8 studies [82,83,84,85,86,87,88,89]). Figure 4 shows how the selected articles are distributed among the different identified tasks.

Furthermore, Table 1 provides a quick overview of the key works we will investigate further in the following sections. The FoG prediction task involving sEMG and ML models is the latest research focus, while tremor analysis has been more extensively studied in past years, particularly in 2019 and the early part of 2020.

### 3.2. Research Questions

This review aims to address the following research questions:

RQ1 What are the main applications of surface electromyography (sEMG) in Parkinson’s disease research?
RQ2 What datasets and acquisition protocols are used in sEMG-based studies for Parkinson’s disease, and how do they impact reproducibility and performance?
RQ3 What preprocessing techniques and feature extraction methods are commonly employed?
RQ4 Which machine learning and deep learning models are most frequently adopted, and how do they compare in terms of performance?
RQ5 What are the main limitations and open challenges in this research field?

These research questions are identified to provide a structured overview of how sEMG signals are combined with ML and DL techniques for Parkinson’s disease analysis, and to identify effective methodological approaches and best practices.

To address RQ1, the reviewed studies are categorized according to their primary application task. RQ2 focuses on the analysis of datasets and acquisition protocols, examining sensor configurations, experimental setups, and their impact on reproducibility and performance. RQ3 investigates the preprocessing techniques and feature extraction methods adopted in each study, while RQ4 analyzes the machine learning and deep learning models employed, with particular attention paid to the reported performance. Finally, RQ5 focuses on identifying the main limitations and open challenges highlighted in the literature.

In the following sections, each selected study is examined in relation to the proposed research questions. The analysis is organized according to the primary application domain (e.g., diagnosis, tremor analysis, and gait analysis), considering the datasets used, preprocessing techniques, machine learning strategies, and reported performance, with the aim of enabling a structured comparison across studies.

## 4. Research Review

### 4.1. Early Diagnosis and Disease Assessment

This section analyzes studies focusing on early diagnosis and disease assessment of Parkinson’s disease using surface EMG signals in combination with ML or DL techniques. A total of six studies were identified within this application domain. For clarity, the selected works are further categorized into two subgroups: (i) studies addressing the classification between Parkinson’s disease patients and healthy control subjects and (ii) studies focusing on the estimation of disease severity and progression. One study addresses both aspects and is therefore discussed in both categories. The analysis follows the research questions defined in Section 3.2, with a particular focus on application tasks (RQ1), methodological approaches (RQ2–RQ4), and reported performance.

#### 4.1.1. sEMG-Based PD Diagnosis

Four studies have investigated the use of sEMG for the early diagnosis of Parkinson’s disease using ML and DL techniques to distinguish patients from healthy controls. Most of these works (Loconsole et al., Adem et al., and Saikia et al. [61,63,64]) rely on time-series data acquired from non-invasive wearable sensors, from which features are either manually extracted and used to train ML models or automatically learned through DL architectures. In contrast, Rezaee et al. [62] employed sEMG spectrograms and deep transfer learning (DTL) to automatically extract high-dimensional feature representations using pre-trained DNNs, followed by feature selection to reduce complexity. While some studies (Adem et al. and Rezaee et al. [62,64]) relied solely on sEMG data, others (Loconsole et al. and Saikia et al. [61,63]) combined it with additional modalities, such as handwriting biometrics or EEG, to improve diagnostic performance. These studies collectively demonstrate the versatility of sEMG-based ML approaches for early PD detection.

The identified works differ in dataset sizes, sensor configurations, and acquisition protocols (Table 2) while sharing the common objective of distinguishing PD from HC. The cohort size ranges from very small (Rezaee et al. [62], eight subjects) to medium (Saikia et al. [63], 60 subjects). When declared, the degree of disease among subjects is consistent in all the articles: generally UPDRS from 2 to 4 and H&Y from 1 to 2. Additionally, sensor types vary from multi-channel sEMG armbands (Loconsole et al. [61]) to laboratory recording systems (Rezaee et al., Saikia et al., and Adem et al. [62,63,64]). The data acquisition protocols also differ; all the authors focused on upper-limb movements. Rezaee et al. [62] acquired signals during 56 days of subjects’ daily life, Loconsole et al. [61] focused on handwriting tasks, Adem et al. [64] analyzed multiple upper-limb movements (e.g., elbow flexion under load, wrist pronation, and shoulder touching), while Saikia et al. [63] considered wrist flexion and extension. The positioning of the sEMG electrodes varies across these studies; only Rezaee et al. [62] declared having followed the SENIAM guidelines, but, overall, the muscle choice is in line with the objective, which is to analyze upper extremities.

The preprocessing strategies, reported in Table 3, are largely consistent across studies and follow standard sEMG processing practices, including bandpass (Saikia et al. [63]) or high/low-pass (Rezaee et al. [62] and Adem et al. [64]) filtering to remove motion artifacts and high-frequency noise. Notch filtering at 50 Hz is commonly applied (Rezaee et al. and Adem et al. [62,64]) to suppress power-line interference. The most prevalent filters are Butterworth filters of various orders, while the notch is typically an IIR filter. However, variability in reporting preprocessing details (e.g., absence of information in [61]) limits reproducibility and comparability across studies. Furthermore, normalization has been performed only by Saikia et al., [63], who applied MVC% (maximum voluntary contraction)-based normalization to compare muscle conditions between various subjects using a grip exercise during the acquisition. In a data preprocessing pipeline aimed at training an ML or DL model, it is also common to apply a windowing strategy to segment the signal. However, for the early diagnosis task, only Rezaee et al. [62] segmented the sEMG data in windows; Loconsole et al. [61] did not declare if they applied a windowing technique, and Saikia et al. and Adem et al. [63,64] did not segment their data but used the signal duration provided by their acquisition protocols. Finally, it is interesting to mention that Adem et al. [64] considered applying data augmentation (from 1000 to 2400 data points) before the preprocessing step, adding white Gaussian noise to the raw signal in order to improve the accuracy and robustness of the classifiers.

Feature extraction strategies, as reported in Table 4, are predominantly based on handcrafted time- and frequency-domain descriptors, with recurrent selection of features such as RMS, MNF, and MDF across studies (Loconsole et al., Saikia et al., Adem et al. [61,63,64]). This consistency suggests that these features capture relevant neuromuscular characteristics for PD discrimination. Feature selection techniques are frequently employed to reduce dimensionality and improve model performance: Loconsole et al. [61] applied principal component analysis (PCA) and observed that RMS and ZC are among the most representative features for PD and HC classification; Adem et al. [64] used the Relief-F algorithm to select the best nine features that line up with those identified by other similar studies. In contrast, Rezaee et al. [62] adopted a data-driven approach using deep transfer learning, where features are automatically extracted from spectrogram representations as input of pre-trained DL networks. They also applied a feature selection algorithm based on merged soft voting. However, while this approach avoids manual feature engineering, it reduces interpretability as the learned features lack direct physiological meaning.

Table 5 shows that, across studies, classical ML models such as SVM, kNN, LDA, and ANN are predominantly used, reflecting the relatively small dataset sizes and reliance on handcrafted features, while DL models are involved through transfer learning approaches where pre-trained networks are used as feature extractors. Model selection appears to be driven more by data characteristics than by task-specific considerations. In particular, in multimodal scenarios, the learning model is trained on fused feature spaces combining the sEMG with auxiliary signals (EEG in [63]; biometric data in [61]). The dataset splitting choice mainly involves train, test, and validation sets, with not substantially different split percentages. Furthermore, a common method used to train ML classifiers is *k*-fold cross-validation. As for the models’ hyperparameters, Saikia et al. and Adem et al. [63,64] did not provide any insights, thus limiting reproducibility. Loconsole et al. [61] used Multi-Objective Genetic Algorithm (MOGA) optimization for ANN hyperparameter tuning, reporting the details of the best architecture. Rezaee et al. [62] trained their model using default parameters. Performance is commonly assessed using accuracy, sensitivity, specificity, F1-score, and AUC (based on confusion matrices). Reported classification accuracies are generally high in controlled settings. However, some works do not report complete performance metrics (Saikia et al. [63]), making their results not comparable to others. Therefore, despite the use of cross-validation techniques to mitigate limited sample sizes, the lack of standardized evaluation protocols and incomplete reporting of performance metrics in some studies hinder fair comparison across approaches.

Overall, sEMG-based approaches for PD diagnosis demonstrate promising performance in controlled experimental settings. However, the significant heterogeneity in datasets, acquisition protocols, preprocessing strategies, and evaluation methodologies limits the comparability of results and hinders generalization. These findings highlight the need for standardized data acquisition and evaluation frameworks to facilitate the clinical translation of sEMG-based machine and deep learning systems.

#### 4.1.2. Disease Severity Assessment Using sEMG

Three studies investigated the use of sEMG signals for Parkinson’s disease severity assessment through machine learning approaches. These works focused on estimating disease severity using either categorical stages or clinical rating scale scores (UPDRS/H&Y), with the aim of supporting clinicians in therapy adjustment and progression monitoring. All three studies relied exclusively on sEMG signals, and none explored multimodal configurations. In addition, all adopted shallow machine learning models rather than deep learning approaches. The main difference between them lies in the target variable: Adem et al. [64] considered categorical severity levels (normal, early, moderate, and advanced), Kleinholdermann et al. [65] predicted UPDRS part III scores during ON and OFF Levodopa states, and Sun et al. [66] estimated H&Y scores. The acquisition tasks also differ: Adem et al. and Kleinholdermann et al. [64,65] analyzed upper-limb muscle activity, whereas Sun et al. [66] focused on lower-limb sEMG during quiet standing based on the hypothesis that inter-limb asymmetry is associated with disease severity.

The studies addressing disease severity assessment using sEMG differ substantially in dataset size, acquisition setup, and motor tasks, as summarized in Table 6. Not all the works focus exclusively on Parkinson’s disease patients as Adem et al. [64] included both healthy controls and PD subjects. However, for the present analysis, only the multiclass severity assessment task was considered, and therefore only the PD subset of that dataset is discussed. The cohort sizes remain limited, ranging from 15 to 45 subjects, and disease severity is represented either categorically or through clinical scales, such as UPDRS part III and Hoehn and Yahr. From an acquisition perspective, the studies employ heterogeneous solutions, ranging from wearable multi-channel armbands to laboratory-grade sEMG systems. Adem et al. and Kleinholdermann et al. [64,65] focused on upper-limb muscle activity during voluntary movements or tapping tasks, whereas Sun et al. [66] analyzed lower-limb activity during quiet standing. This variability reflects different clinical hypotheses and also limits direct comparability between studies.

The preprocessing pipelines used in severity assessment studies generally follow standard sEMG conditioning practices, although relevant differences remain (Table 7). Not all the studies applied both high-pass and low-pass filtering or an equivalent bandpass filter. For example, Kleinholdermann et al. [65] only applied high-pass filtering, while Sun et al. [66] did not filter the signal within the typical sEMG frequency range. A common step is the use of a 50 Hz notch filter to suppress power-line interference. Additional preprocessing choices also vary; Sun et al. [66] applied detrending, rectification, and amplitude normalization to extract the signal amplitude, whereas Adem et al. [64] introduced raw data augmentation by adding white Gaussian noise to improve the robustness of the classifier. Signal segmentation strategies also depend on task duration and analysis objectives: short and well-defined tasks, such as tapping, are segmented into fixed-length windows ([65]), while longer static tasks, such as standing, are analyzed as continuous recordings ([66]). This lack of standardized preprocessing and segmentation protocols introduces additional variability in signal representation and may affect model robustness, performance, and generalizability.

All the studies relied on handcrafted feature extraction to characterize PD severity (see Table 8). The extracted features are mainly drawn from the time and frequency domains to capture changes in muscle activation amplitude, variability, and spectral distribution associated with disease progression. Among them, RMS is the only feature shared across all the studies, suggesting that it represents one of the most robust descriptors for this task. Feature selection was applied only by Adem et al. [64], who used the Relief-F algorithm to reduce the initial feature set from 14 to the nine most informative descriptors. Sun et al. [66], on the other hand, proposed a hybrid strategy based on Fisher vectors to combine signal-level information with handcrafted features, arguing that conventional approaches often rely on either the raw signal or extracted features alone. Despite the variability in the selected characteristics, the extracted features generally retain clinical interpretability, reflecting aspects such as muscle activation intensity, variability, and rhythm. Notably, none of the studies explored automatic or deep feature learning, likely due to the limited dataset sizes and the relatively small scale of the available cohorts.

As shown in Table 9, PD severity assessment using sEMG has been predominantly approached with shallow machine learning models, including SVM, kNN, LDA, DT, and RF. Model selection appears to be driven more by dataset size and feature dimensionality than by task-specific considerations, and deep learning approaches are notably absent in this application domain. Performance evaluation strategies vary substantially across studies, reflecting differences in target variables and problem formulation. In particular, Kleinholdermann et al. [65] assess performance using the correlation between true and predicted UPDRS scores but do not report additional metrics that would better characterize predictive reliability and generalization. Although the reported results suggest promising predictive potential, direct comparison remains difficult due to heterogeneous evaluation metrics, limited cohort sizes, and incomplete reporting of training and validation procedures. These factors underline the need for standardized benchmarks and evaluation protocols in future sEMG-based studies on PD severity assessment. It is also worth noting that Kleinholdermann et al. [65] are the only authors in this subgroup to have made their code publicly available [92], partially supporting reproducibility.

In conclusion, sEMG-based approaches for severity assessment show promising potential in capturing clinically relevant neuromuscular alterations. However, the current studies are limited by small cohort sizes, reliance on handcrafted features, and the exclusive use of shallow machine learning models. Moreover, the significant heterogeneity in acquisition protocols, preprocessing strategies, and evaluation methodologies prevents reliable comparison and limits generalization. These findings highlight the need for larger standardized datasets and more robust evaluation frameworks, as well as the exploration of advanced data-driven approaches, to enable clinical translation of sEMG-based severity assessment systems.

### 4.2. Differential Diagnosis

Among the selected papers, four studies investigate the use of sEMG data in combination with other sensing modalities for differential diagnosis tasks involving Parkinson’s disease. Three works [67,68,69] focus on distinguishing PD from essential tremor (ET) using signals acquired from the upper limbs, while Fricke et al. [70] address the classification of gait disorders using sEMG and inertial data from the lower limbs. Although the latter does not explicitly frame the problem as a differential diagnosis between PD and other diseases, it includes a multi-class classification of normal, hypokinetic, and ataxic gait, where hypokinetic gait is strongly associated with PD. For this reason, the study is considered relevant in this context, and only the three-class classification task is analyzed. A common characteristic across all the studies is the adoption of a multimodal approach, combining sEMG with accelerometer data. However, the relative contribution of each modality to the final performance is not consistently evaluated, limiting the interpretability of multimodal fusion strategies.

Table 10 summarizes the dataset characteristics and acquisition protocols. Compared to previously discussed tasks, these studies generally involve larger cohorts. In particular, the work of Xing et al. [68] represents the largest study on PD versus ET identification using sEMG and accelerometry. Sil et al. [69] further strengthen this trend by including an external validation dataset (20 PD, 20 ET) acquired under different clinical conditions and using different devices, demonstrating improved generalizability and robustness. The sensor configurations are relatively consistent across studies, with Tang et al. and Xing et al. [67,68] employing the same acquisition system. All the studies combine sEMG and accelerometer data, either for multimodal classification [67,68,69] or signal segmentation purposes, as in Fricke et al. [70], where inertial data are used to identify gait cycles and segment sEMG signals accordingly. Muscle selection is consistent in PD versus ET studies [67,68,69], focusing on flexor and extensor muscles of the forearm, while Fricke et al. [70] analyzed multiple lower-limb muscles (rectus femoris, vastus medialis, biceps femoris, tibialis anterioris, and gastrocnemius lateralis) to characterize gait patterns. Acquisition protocols for PD and ET differential diagnosis studies involve multiple postural conditions with slight variations (e.g., inclusion of weights), whereas Fricke et al. [70] employed functional tasks, such as the timed up and go (TUG) test, including walking, turning and tandem gait.

Preprocessing and windowing strategies are relatively consistent across studies, although there is some variability (Table 11). In general, normalization (e.g., Z-score) [67,68], rectification [69,70], and basic filtering operations [68,70] are applied. However, only Fricke et al. [70] adopted a conventional sEMG filtering pipeline (high-pass, low-pass, and rectification), while other works employed simplified or task-specific preprocessing strategies. For example Xing et al. [68] preprocessed their signal differently based on the classifier method (ML or DL).

Notably, none of the studies applied conventional fixed-window segmentation. Instead, signals are processed as continuous recordings or segmented based on task-specific events. For example, Fricke et al. [70] used accelerometer data to identify gait cycles and segment sEMG signals accordingly, followed by resampling to ensure uniform signal length across subjects. This event-driven segmentation reflects the nature of the analyzed tasks but introduces variability in signal representation due to the different durations of the subjects’ gait cycles.

Feature extraction approaches vary between handcrafted and automatic methods, as shown in Table 12. Handcrafted features are widely used, particularly in studies employing traditional machine learning models (Xing et al., Sil et al., and Fricke et al. [68,69,70]). These features are mainly derived from time, frequency and power domains, capturing relevant disease characteristics, such as dominant frequency and signal amplitude. Xing et al. [68] also investigated the inclusion of two demographic features (age and sex), which, however, did not show any relationship with a correct identification of ET or PD. In contrast, Tang et al. and Fricke et al. [67,70] employed deep learning approaches for automatic feature extraction. Tang et al. [67] proposed a multimodal fusion architecture based on cross-attention and CNN (MFCA-Net), demonstrating improved robustness compared to simple feature concatenation. Fricke et al. [70] converted sEMG signals into time–frequency representations using continuous wavelet transform (CWT) for CNN-based classification while also extracting handcrafted features for traditional ML models. Feature selection is commonly applied to reduce dimensionality and improve model performance. Xing et al. [68] evaluated feature importance based on effect size, Sil et al. [69] employed RFECV combined with SHAP analysis, and Fricke et al. [70] applied PCA to retain components explaining 99% of variance. These approaches highlight the importance of identifying discriminative features while mitigating redundancy and overfitting. The last column of Table 12 reports only the best features obtained from the sEMG signal.

As shown in Table 13, both traditional ML models and DL architectures are adopted, with no clear dominance of one paradigm. Ensemble methods, particularly XGBoost, achieve strong performance in multiple studies [68,69], while CNN-based models demonstrate competitive results in multimodal and time–frequency settings [67,70]. All the studies used cross-validation strategies, including standard split CV (Tang et al. [67]), k-fold (Xing et al. [68]) and nested cross-validation (Sil et al. [69] employed an inner CV for feature selection and hyperparameter tuning and an outer CV for performance evaluation), while Fricke et al. [70] adopted a leave-one-subject-out (LOSO) approach to better assess subject-independent performance. As for the metrics used to evaluate performance, most studies consider confusion matrix, accuracy, precision, recall, F1-score, ROC, and AUC-ROC. To better compare the models, Table 13 reports the classification accuracy as it is a common metric provided across all the reviewed articles. Despite generally high reported accuracies, differences in evaluation protocols and incomplete reporting of hyperparameters in some studies ([67,70]) limit reproducibility and fair comparison. Additional analyses highlight the benefits of multimodal approaches. Tang et al. [67] show that combining sEMG and accelerometer data improves performance by approximately 13% compared to single-modality configurations. Sil et al. [69] identify tremor-related features at rest as the most discriminative for PD versus ET classification.

Overall, PD differential diagnosis studies consistently rely on multimodal approaches combining sEMG and inertial data, leveraging the complementary information provided by muscular and kinematic signals. While both ensemble machine learning models and deep learning architectures achieve high classification performance, no clear consensus emerges regarding the optimal modeling strategy, suggesting strong dependence on dataset characteristics and experimental design. Despite promising results, the current literature is constrained by variability in acquisition protocols, feature extraction strategies, and evaluation methodologies, which limits reproducibility and comparability across studies. Furthermore, although the dataset sizes are larger than in other tasks, issues such as class imbalance and limited diversity persist. Addressing these challenges through standardized datasets, benchmarking protocols, and systematic evaluation of multimodal contributions will be essential for advancing the clinical applicability of sEMG-based differential diagnosis systems.

### 4.3. Freezing of Gait Prediction

Freezing of gait (FoG) is one of the most disabling motor symptoms of PD, characterized by brief episodes in which patients are unable to initiate or continue walking [93]. These events are particularly frequent when navigating narrow spaces, turning, or approaching obstacles, and they increase the risk of falls while also contributing to anxiety and fear of movement. Continuous monitoring of FoG episodes is therefore essential for timely intervention, and wearable sensors combined with advanced machine learning approaches offer a promising solution [94]. Multiple sensing modalities, including EEG, sEMG, accelerometers (ACCs), and skin conductance (SC), have been explored for this purpose, with sEMG providing direct information on the muscular activation disturbances associated with FoG events. Five studies were identified in this review that specifically investigated wearable sensor-based FoG detection or prediction using ML and DL techniques. All of them are based on the same publicly available multimodal dataset [95], which includes sEMG, among other physiological and kinematic signals. The studies differ slightly in their objectives. Zhang et al. released the dataset, and Murtaza et al. and Gupta et al. [71,72,73] focused on identifying the most effective sensor configuration and classification models for FoG detection or early prediction. In contrast, Munjal et al. and Hou et al. [74,75] concentrated on developing lightweight models for real-time inference on low-power edge devices.

Since all five studies rely on the same database, the dataset is described once here based on the acquisition protocol presented by [71]. The “Multimodal Dataset of Freezing of Gait in Parkinson’s Disease” [95] was, at the time of publication, the first publicly available multimodal dataset specifically designed for FoG analysis in PD. Data collection was performed in the off-medication state to maximize the probability of recording FoG episodes. Eighteen patients were initially enrolled, but only 12 valid acquisitions were retained, and only 10 of these subjects exhibited a substantial number of FoG events. Signals were acquired using a 32-channel wireless MOVE system ($f_{s}=1000$ Hz) for EEG (25 channels) and sEMG (three channels), together with a TDK MPU6050 six-DoF sensor ($f_{s}=500$ Hz) for accelerometry and skin conductance. The sEMG electrodes were placed on the gastrocnemius (GS) of the right leg and on the tibialis anterioris (TA) of both legs, while accelerometers were positioned on both lateral tibias, the lumbar region, and the left arm. The protocol consisted of two TUG-based tasks designed to trigger FoG episodes, including sitting, standing, straight walking, turning, passing through narrow corridors, bypassing obstacles, and turning in restricted spaces. Although the acquisition protocol is shared, the reviewed studies differ in the subset of patients and sensing modalities selected for analysis, as shown in Table 14. Most studies used the full 12-subject cohort, while Gupta et al. [73] restricted the analysis to the 10 subjects with the highest number of FoG episodes. Similarly, some studies used all the available modalities [71,72], whereas others focused on smaller subsets of sEMG, ACC, EEG, and SC signals [73,74,75].

Preprocessing and windowing strategies are relatively consistent across FoG studies, as summarized in Table 15. Because the dataset contains signals acquired at different sampling frequencies, resampling to a common frequency is a standard step in most studies. Typical sEMG preprocessing includes bandpass filtering and, in some cases, notch filtering to suppress power-line interference. Hou et al. [75] are the only authors who do not clearly report the adopted preprocessing steps, thus reducing reproducibility. Signal segmentation is essential in FoG detection, and all the studies perform window-based analysis. A window length of 3 s is the most common choice and is in line with the broader FoG literature. Gupta et al. [73], however, used shorter windows for early prediction, labeling segments occurring immediately before an FoG event as positive samples. Notably, Murtaza et al. [72], with the aim of surpassing Zhang et al.’s [71] performance, tested different windowing choices in order to identify the most suitable one for this dataset. Window-labeling strategies vary: Zhang et al. and Murtaza et al. [71,72] relied on the percentage of FoG (PFG) points within each window, using threshold-based labeling, while Munjal et al. [74] adopted majority voting. Hou et al. [75] did not declare the labeling technique. These differences in segmentation and labeling have a direct impact on class balance and model performance.

Feature extraction strategies are more homogeneous than in other application domains, as reported in Table 16. Studies based on traditional ML models (Zhang et al., Murtaza et al., and Munjal et al. [71,72,74]) generally rely on the same handcrafted feature set, consisting of mean absolute value (MAV), zero crossing rate (ZC), slope sign change (SSC), and waveform length (WL). These time-domain descriptors are computationally simple and well suited for real-time applications, which partly explains their repeated adoption in FoG studies. Gupta et al. and Hou et al. [73,75] also consider DL approaches and therefore employed automatic feature extraction. Gupta et al. [73] compared handcrafted and automatically learned features, although the extracted descriptors are only described generically as 23 time-domain features, limiting interpretability. Notably, none of the reviewed studies explored feature selection techniques despite the clear relevance of dimensionality reduction for multimodal data. This represents one of the most evident methodological gaps in the current literature.

Model development in FoG studies is strongly oriented toward identifying the best sensor combination and the most effective model configuration (Table 17). Cross-validation is the most common validation strategy, while LOSO is performed by Gupta et al. and Hou et al. [73,75] to better assess inter-subject generalization. Three studies (Zhang et al., Murtaza et al., and Gupta et al. [71,72,73]) compared traditional ML models, with Gupta et al. [73] additionally evaluating a DL architecture (1D CNN–LSTM). Zhang et al. [71] report strong performance using an SVM with RBF kernel, while Murtaza et al. [72] achieve even higher accuracy by optimizing PFG thresholds and addressing class imbalance with SMOTE. Gupta et al. [73] show that a 1D CNN–LSTM model can achieve performance comparable to shallow ML approaches while enabling early prediction of FoG events. Munjal et al. and Hou et al. [74,75] focused instead on tinyML-oriented DNN models for edge deployment. Both investigated post-training quantization and pruning, showing that lightweight models can still retain competitive performance for real-time inference on low-power devices. These studies are particularly relevant from a translational perspective as they move beyond offline classification and toward deployable FoG monitoring systems. Another key result shared across the reviewed studies is the consistent advantage of multimodal over unimodal configurations. When explicitly compared, models combining sEMG with inertial and/or EEG features outperform single-modality approaches, reinforcing the idea that FoG is best characterized through complementary neuromuscular and kinematic information. We consider it important to mention that, in order to facilitate reproducibility, Gupta et al., Munjal et al., and Hou et al. [73,74,75] accurately described the architecture of the models implemented.

To sum up, FoG detection and prediction studies based on sEMG show a relatively high degree of methodological consistency, largely because they rely on the same publicly available multimodal dataset. This has enabled direct comparison of multiple ML and DL approaches, highlighting the importance of window-based analysis, multimodal fusion, and labeling strategies. Specifically, the use of multimodal features enables achieving higher and more reliable performance; the optimal window length is 3 s, with a varying overlap percentage; PFG-based labeling with higher thresholds is the preferred solution. Among the reviewed methods, SVM-based models remain strong baselines for FoG detection, while CNN–LSTM and lightweight DNN architectures show promising potential for early prediction and real-time on-device inference. At the same time, the current literature remains constrained by the dependence on a single dataset, limited sample size, class imbalance, and the absence of systematic feature selection strategies. These limitations restrict generalizability and make it difficult to assess transferability across cohorts and recording conditions. Future work should therefore focus on collecting larger and more diverse datasets, exploring balancing and feature selection techniques, and validating deployable models across independent populations to support the clinical adoption of sEMG-based FoG monitoring systems.

### 4.4. Tremor Prediction and Quantification

Tremor is among the most prevalent motor symptoms of Parkinson’s disease, affecting approximately 79–90% of patients. It consists of involuntary rhythmic movements of one or more body parts and is commonly classified into resting and action tremor depending on whether the involved muscles are relaxed or active [96]. Tremor substantially interferes with daily activities and quality of life, making its objective assessment essential for treatment selection and disease monitoring. In addition to its diagnostic and clinical relevance, tremor analysis is also important for evaluating assistive and non-pharmacological interventions aimed at symptom suppression. Beyond dopaminergic therapy, several non-drug strategies have been explored for tremor reduction. Deep brain stimulation (DBS) is an effective but invasive neuromodulation technique requiring surgical implantation. Functional electrical stimulation (FES) represents a less invasive alternative based on surface stimulation of muscles to support motor control [97]. Biofeedback has also been investigated as a non-invasive therapeutic strategy, typically relying on visual or auditory cues to help patients modulate tremor-related activity [98]. Six studies were identified that focused on tremor analysis in PD using sEMG signals. These works can be broadly divided into two groups: studies aimed at tremor identification and quantification for diagnostic and monitoring purposes (Lin et al., Qin et al., and Farhani et al. [79,80,81]) and studies evaluating tremor characteristics to assess suppression strategies, such as DBS, biofeedback, or FES (Wang et al., Davoudi et al., and Zanini et al. [76,77,78]). Farhani et al. [81] partially overlap both categories as they classify tremor type and task with the long-term goal of improving wearable robotic tremor suppression systems. From a modeling perspective, Wang et al. and Davoudi et al. [76,77] rely mainly on ML approaches, whereas Zanini et al., Lin et al., Qin et al., and Farhani et al. [78,79,80,81] explore DL architectures for PD tremor prediction and classification.

Table 18 summarizes the datasets and acquisition protocols adopted in tremor-related studies. In contrast to the FoG literature, no common public dataset is available for this task; each study relies on privately collected data, or, in the case of Zanini et al. [78], on data provided by a previous private study. As a result, cohort size and patient representation vary substantially across works. Qin et al. [80] report the largest cohort and are also the only authors to include all five UPDRS tremor levels, while Zanini et al. [78] analyze the smallest sample. Regarding disease severity, Wang et al., Davoudi et al. and Lin et al. [76,77,79] include subjects with relatively similar and generally mild-to-moderate tremor severity, whereas Zanini et al. and Farhani et al. [78,81] do not report UPDRS or H&Y values. This heterogeneity makes direct comparison across studies difficult and limits the interpretability of reported performance. The type of tremor analyzed also differs. Wang et al., Davoudi et al., and Zanini et al. [76,77,78] focus specifically on resting tremor (RT), while Lin et al. and Qin et al. [79,80] study upper-limb tremor without clearly specifying the subtype. Farhani et al. [81], with the objective of classifying the type of tremor, explicitly address resting, postural, and action tremor. Most studies rely exclusively on sEMG, with Lin et al. [79] representing the only multimodal approach by combining sEMG with inertial signals. The monitored muscles are predominantly located in the upper limbs, especially forearm and wrist muscles, although Wang et al. [76] also include lower-limb muscles to assess DBS effects. Overall, acquisition tasks are largely based on stationary postures and controlled arm or hand movements, reflecting the need to isolate tremor-related activity.

The preprocessing and segmentation strategies adopted in tremor-related studies are summarized in Table 19. As in other sEMG applications, filtering is the most common preprocessing step, but the selected frequency ranges are more heterogeneous because tremor emphasizes oscillatory activity in lower frequency bands (around 4 to 6 Hz). While general sEMG analysis often considers broader bands, tremor-focused works use narrower filters (200 Hz, 100 Hz or even 20 Hz, respectively, in Wang et al., Davoudi et al., Farhani et al. and [76,77,81]) tailored to the expected tremor frequency content. Window-based segmentation is applied in all the studies, reflecting the time-varying nature of tremor signals and the need to capture short-term oscillatory behavior. However, window length varies widely, from 250 ms to 3 s, depending on the study objective. Short windows are generally preferred for prediction [78] or type classification [81], whereas longer windows are used in monitoring or therapy-evaluation settings [77,79]. Only Farhani et al. [81] explicitly reported overlap between adjacent windows, whereas Qin et al. [80] did not report preprocessing details, which limits reproducibility. Zanini et al. [78] provided an interesting comparison between preprocessed sEMG and a smoothed and scaled version of the raw signal, allowing an indirect assessment of how strongly model performance depends on preprocessing.

Feature extraction strategies are highly task-dependent in tremor studies (Table 20). Handcrafted features are used in only a minority of works, mainly when the adopted model requires explicit descriptors or when the goal is to maintain physiological interpretability. Wang et al. [76] extracted interpretable biomarkers, such as weighted RMS and spectral peak measures, to evaluate DBS efficacy, while Qin et al. [80] computed statistical and spectral descriptors to support a CNN-based similarity learning framework. In contrast, Davoudi et al. [77] employed an unsupervised clustering strategy and therefore do not define a conventional feature extraction step. The features chosen by feature selection algorithms, such as statistical tests in Wang et al. and Farhani et al. [76,81], align closely with sEMG features already discussed in previous sections. Deep learning-based studies rely primarily on automatic feature learning; Zanini et al., Lin et al., and Farhani et al. [78,79,81] used neural architectures to learn discriminative tremor representations directly from the signal. Among these, Lin et al. [79] proposed one of the most structured architectures, combining fully convolutional layers for spatial feature extraction, LSTM units for temporal modeling, and an attention mechanism to enhance discriminative information. Overall, this confirms that tremor analysis is one of the application areas where automatic feature learning is explored more actively than in other PD tasks.

Table 21 summarizes the adopted training strategies, models, and reported performance. The heterogeneity of the study objectives is reflected in the diversity of the modeling approaches. Wang et al. [76] used Gaussian process regression (GPR) to predict DBS efficacy, reporting a correlation with clinical improvement rather than conventional classification metrics. Davoudi et al. [77] adopted an AdaBoost-based clustering framework to investigate the effects of auditory biofeedback on tremor patterns, obtaining qualitative rather than directly comparable quantitative results. Zanini et al. [78] proposed a deep learning framework for anticipating resting tremor patterns in support of FES control, evaluating performance through a custom error function γ rather than standard diagnostic metrics. In contrast, Lin et al., Qin et al., and Farhani et al. [79,80,81] addressed more conventional predictive tasks and report comparable classification performance. Lin et al. [79] targeted mild tremor detection using a hybrid LSTM–FCN architecture with attention; Qin et al. [80] reformulated tremor severity estimation as a similarity learning problem using a dedicated CNN (SNet); Farhani et al. [81] employed a BiLSTM combined with neural architecture search (NAS) to classify tremor type and task. Across these studies, deep learning models consistently achieve strong performance, suggesting that tremor-related sEMG patterns are particularly suitable for data-driven modeling. However, comparison across studies remains difficult because performance is reported using different metrics and because objectives range from symptom quantification to therapy evaluation and control-oriented prediction. Nevertheless, a recurring trend is that multimodal input, when available, tends to improve discriminative performance, as suggested by the slightly stronger results reported by Lin et al. [79] compared with purely sEMG-based approaches.

In conclusion, tremor-related studies represent one of the most heterogeneous application areas of sEMG-based analysis in PD, spanning symptom detection, severity quantification, tremor-type classification, and evaluation of suppression strategies. Despite this variability, the reviewed works consistently show that tremor characteristics can be effectively captured from sEMG signals, both for clinical assessment and for the development of assistive or therapeutic systems. Compared with other application domains, tremor analysis shows stronger adoption of deep learning approaches, which appear to be particularly well suited to modeling the temporal and oscillatory structure of tremor-related sEMG signals. However, the current literature is constrained by the absence of shared public datasets, limited sample sizes ([77,78,81]), incomplete reporting of clinical severity, and the frequent focus on restricted settings, such as resting tremor ([76,77,78]) or unilateral upper-limb measurements ([79,81]). These limitations reduce reproducibility and hinder generalization. Future work should therefore prioritize more standardized and diverse datasets, clearer clinical characterization of cohorts, and broader evaluation across tremor subtypes and recording conditions.

### 4.5. Gait Analysis and Motor Symptom Quantification

This section groups studies that investigate the use of sEMG signals and artificial intelligence techniques for PD characteristics that do not fall directly within the previously analyzed tasks of diagnosis, differential diagnosis, freezing of gait, or tremor analysis. These works mainly address gait analysis and the quantification of motor symptoms such as bradykinesia and rigidity. PD commonly affects gait and movement through symptoms such as bradykinesia, rigidity, postural instability [24], dystonia, insufficient leg strength (ILS), and freezing of gait (FoG) [23]. Bradykinesia, defined as slowness of movement, is one of the most disabling manifestations of PD and strongly affects quality of life [48]. Rigidity is characterized by increased muscle tone and resistance to passive limb movements, resulting in reduced flexibility and coordination [99]. Dystonia and insufficient leg strength further alter lower-limb function and gait [23,84]. In this context, gait analysis is a key component of PD and is increasingly performed using wearable sensors, which provide a markerless, non-invasive, and low-cost solution. While inertial measurement units (IMUs) are widely used to detect gait events and segment gait cycles, they do not directly capture neuromuscular activity. Therefore, sEMG represents a valuable complementary or alternative modality for characterizing pathological gait.

Eight studies were identified in this application domain. Lin et al. and Kozulin et al. [82,83] focused on bradykinesia severity estimation, while Alves et al. [85] investigated rigidity quantification through statistical analysis of sEMG signals. Although the latter study does not evaluate a machine learning model, it is included because it highlights the potential of sEMG as a quantitative biomarker for future ML- or DL-based rigidity assessment. Guo et al. [84] used ML to classify different gait disorders, including rigidity, dystonia, insufficient leg strength, and FoG. Liu et al. [86] developed a DL framework for automatic gait scoring in PD. Three additional studies addressed sEMG signal segmentation or gait event detection: Haufe et al. [87] predicted gait events directly from sEMG using an ML model, while Bengacemi et al. (2021, 2024) [88,89] investigated activity segmentation and later extended the problem to joint segmentation and PD classification.

Table 22 summarizes the dataset characteristics and acquisition protocols. Across these studies cohort sizes remain limited: Lin et al. [82] report the largest sample, although their dataset is imbalanced in favor of PD subjects; Guo et al. [84] also include a relatively larger cohort, again with an uneven PD–control ratio. When clinical severity is declared, most studies cover only mild-to-moderate disease stages, typically within UPDRS or H&Y levels of 0–3, leaving severe stages largely unexplored. Most studies rely primarily on sEMG data; however, Lin et al., Guo et al., and Haufe et al. [82,84,87] also integrate inertial data, either to improve predictive performance or to segment sEMG recordings into gait cycles. A clear distinction emerges between upper-limb and lower-limb acquisitions. Studies on bradykinesia and rigidity assessment [82,83,85] focus on forearm and wrist muscles during controlled hand movements, whereas gait-related works [84,86,87,88,89] analyze lower-limb muscles during walking tasks. This division reflects the symptom-specific role of sEMG in PD assessment.

Preprocessing and segmentation choices are reported in Table 23. Compared with other application domains, the level of detail is generally weaker in this subgroup. Several studies do not provide sufficient detail on preprocessing pipelines, and some also omit a clear description of the adopted windowing strategy. In particular, Lin et al. [82] do not report either preprocessing or segmentation details, while Guo et al. and Bengacemi et al. (2021, 2024) [84,88,89] specify window lengths but not the preceding signal processing steps. Among the studies that do describe their pipelines, the selected operations are consistent with standard sEMG practice, including bandpass filtering, notch filtering, rectification, envelope extraction, and normalization. However, the normalization strategies vary substantially. Alves et al. [85] used maximum voluntary contraction (MVC)-based normalization; Haufe et al. [87] applied percentile-based scaling; Liu et al. [86] mention normalization without describing the adopted procedure. Segmentation is similarly heterogeneous: some studies adopt fixed-length windows, others use gait cycles, and Bengacemi et al. (2021, 2024) [88,89] explicitly optimized window size experimentally. These differences introduce variability in the resulting signal representation and reduce methodological comparability. It is also worth noting that Lin et al. [82] applied an LSTM–VAE strategy to augment minority samples and address class imbalance.

Feature engineering strategies are reported in Table 24. Handcrafted feature extraction remains the dominant approach, although some studies integrate automatic feature learning. Lin et al. [82] combined automatically extracted features from video-based information (using FCNN + LSTM) with handcrafted features derived from IMU and sEMG data. Liu et al. [86] relied on automatic feature learning through a multi-branch deep architecture that processes sEMG envelopes, sEMG wavelet representations and IMU-derived gait parameters. The remaining studies are mainly based on handcrafted descriptors. In particular, Guo et al. [84] compared features extracted from sEMG, IMU, and plantar pressure signals, concluding that sEMG features are the most informative for distinguishing gait disorders. Bengacemi et al. (2021, 2024) [88,89] focused on wavelet-extracted features and identify log wavelet energy (LWE) as the most representative descriptor for sEMG activity segmentation. Kozulin et al. [83] compared reduced and expanded handcrafted feature sets for bradykinesia estimation, showing that the broader set provides better reliability. Furthermore, Lin et al. [82] employed a ResNet to reduce the amount of features in order to limit redundancy, which could lower the model’s performance and lead to overfitting. Overall, this subgroup confirms the continued relevance of handcrafted features for gait and symptom analysis, especially when datasets are small and clinical interpretability is important.

The adopted training strategies, models and final performance metrics are summarized in Table 25. Subject-wise validation is common in this subgroup, with LOSO cross-validation frequently used to better assess generalization to unseen subjects. Kozulin et al. and Liu et al. [83,86], in their implementation of LOSO, made sure to include all the categories in the test set (ON, OFF, and HC for the former; two HCs and one PD per UPDRS level for the latter). Two studies, Lin et al. and Liu et al. [82,86], developed deep learning models for severity estimation. Lin et al. [82] proposed a hybrid LSTM–FCN architecture that combines skeleton and inertial + sEMG features, showing that multimodal fusion, feature shrinking, and data balancing improve performance. Similarly, Liu et al. [86] used a multi-branch convolutional framework for automatic PD gait scoring, combining sEMG and IMU information to emulate the multidimensional scoring process of clinicians: after segmenting the sEMG signal in gait cycles using the IMU, each branch takes sEMG envelope, wavelet or IMU-extracted gait parameters. The outputs are fused together to get an overall UPDRS score. The remaining studies mainly investigate shallow ML models. Alves et al. [85] do not perform predictive modeling, but their correlation and ANOVA analyses indicate that sEMG may serve as a valid quantitative biomarker for rigidity assessment. Kozulin et al. [83] show that random forests perform well for bradykinesia severity estimation, although their results differ depending on the movement task. Guo et al. [84] also identify random forests as the best-performing ML model for PD gait disorder classification; Haufe et al. [87] demonstrate that gait events can be reliably estimated directly from sEMG without relying on IMUs, with vastus lateralis (VL) emerging as the most informative muscle. Finally, Bengacemi et al. (2021, 2024) [88,89] show that hidden Markov models (HMMs) can effectively support both activity segmentation and, in the extended version, joint segmentation and PD classification. Despite promising results, reporting quality varies considerably. Several studies do not fully describe their hyperparameter choices, data splits, or validation details, which limits reproducibility and hinders fair comparison across approaches. In this respect, Kozulin et al. [83] stand out as the only authors in their subgroup to share both the dataset and code. By contrast, Haufe et al. [87] report code availability, but the referenced repository appears to be empty.

Overall, this subgroup highlights the versatility of sEMG for characterizing PD motor symptoms beyond tremor and freezing of gait, including bradykinesia, rigidity, gait disorders, and gait event timing. The reviewed studies suggest that sEMG can support both symptom quantification and gait analysis, and in some cases may reduce the need for purely IMU-based detection by providing direct neuromuscular information. At the same time, this is also one of the most heterogeneous application domains in the literature, both in terms of clinical targets and methodological approaches. Small datasets, limited disease severity coverage, class imbalance, and incomplete methodological reporting remain the main limitations. Future work should prioritize more standardized protocols, larger and better-balanced cohorts, clearer reporting practices, and broader evaluation of multimodal approaches in order to strengthen the clinical applicability of sEMG-based systems for gait and motor symptom assessment in Parkinson’s disease.

## 5. Discussion

The studies reviewed in the previous sections explored the use of surface electromyography (sEMG) combined with machine learning and deep learning techniques for the analysis of Parkinson’s disease across different application tasks. In this section, we discuss the main findings of the review process, which are directly linked to the research questions defined in Section 3.2. Specifically, RQ1 is related to the main applications of sEMG in PD research; RQ2 addresses datasets and acquisition protocols; RQ3 focuses on preprocessing and feature extraction techniques; RQ4 evaluates which ML and DL techniques are most frequently adopted and the performance they achieve; RQ5 investigates the main limitations and open challenges in this research field.

With respect to RQ1, the reviewed papers were grouped into the following categories: disease diagnosis and assessment, differential diagnosis to distinguish PD from other similar neurological disorders, freezing of gait detection and prediction, tremor detection and quantification, other motor symptoms’ severity prediction, and gait analysis. Although these studies share a common objective, which is extracting useful and representative information on PD from sEMG acquisitions, they differ in terms of dataset characteristics, acquisition protocols, signal processing pipelines, and adopted machine learning methodologies. These differences can influence the achieved results and their generalizability, highlighting the importance of a systematic analysis of each study’s experimental design. Therefore, this section discusses the reviewed studies by focusing on three main aspects: datasets and acquisition protocols (Section 5.1), signal processing and feature extraction strategies (Section 5.2), and machine and deep learning models and their performance (Section 5.3). Finally, Section 5.4 discusses the main limitations that currently hinder the clinical validation of machine learning strategies for sEMG data analysis and outlines possible future developments.

### 5.1. Datasets and Acquisition Protocols

This section addresses RQ2 to identify the most commonly used datasets and acquisition protocols in sEMG-based studies for Parkinson’s disease. It also analyzes how their variability impacts reproducibility and model performance. Dataset characteristics and acquisition protocols are critical factors influencing the performance, reliability, and generalizability of ML models. The reviewed studies differ in terms of dataset size, sensor configuration, monitored muscles, recording conditions, and experimental tasks used to trigger tremor events or gait patterns. An in-depth analysis of these aspects allows a better understanding of the reliability of the obtained results and comparability among studies.

From a dataset perspective, the most variable characteristic is size, which is both category- and study-dependent. In Table 26, we report the mean value ± standard deviation of dataset size for each category. The last row indicates the overall variability across all the considered papers. These measures confirm the high variability that characterizes PD studies involving sEMG acquisitions.

**Figure 5:**
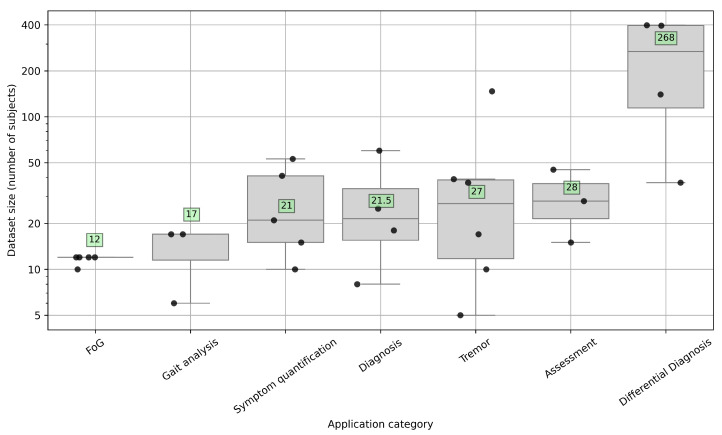
Box plot of the dataset size (number of subjects involved in the studies) across the different application categories in which the sEMG signal has been used. Black dots indicate the position of each study. Green rectangles show the median value for each category. To provide better visualization, given the large difference between the minimum (5) and maximum (398) values, the y-axis is shown on a log scale.

These differences in sample size can also be observed in Figure 5, where common size ranges as well as minimum and maximum values can be identified. Furthermore, the figure highlights which category includes the most populated datasets. In particular, most applications rely on datasets with fewer than 50 subjects, whereas differential diagnosis studies tend to use substantially larger cohorts. The smallest dataset corresponds to the study by Zanini et al. [78], who used the dataset collected by Pinheiro et al. [97], comprising only five subjects divided into four PD and one ET for resting tremor prediction. On the other hand, the largest dataset was reported by Xing et al. [68] with 398 subjects (257 PD, 141 ET) collected for differential diagnosis between PD and ET. This is followed by Sil et al. [69], who acquired sEMG data from 396 patients (124 PD, 272 ET) for the same task. As mentioned in the dedicated section, the box plot also highlights that all five FoG prediction studies analyzed the same dataset, therefore sharing the same cohort size (12 patients), except for Gupta et al. [73], who considered only the 10 patients with the highest number of FoG occurrences.

**Figure 6:**
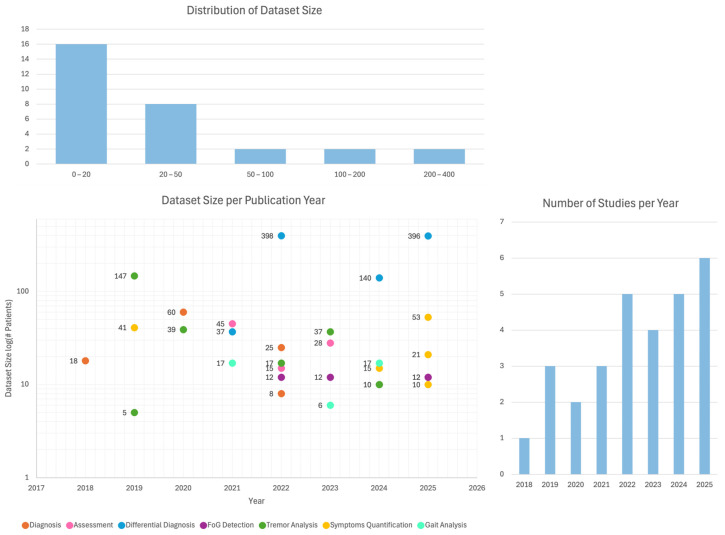
The scatter plot describes the distribution of dataset sizes over the years, with different colors representing different tasks. The upper bar plot shows how many papers fall within a given range of participant numbers. The bar plot on the right shows the number of studies per year regardless of the category.

Figure 6 shows the distribution of dataset sizes across studies over the years. From the scatter plot, it can be observed that most datasets contain fewer than 30 subjects, highlighting the limited scale of many sEMG studies. Although a slight increase in dataset size can be observed in more recent works, large-scale datasets remain relatively uncommon, and only a few studies exceed 100 participants. The bar plot above the scatter plot provides another representation of the number of studies using a given number of participants: the majority fall in the 0–20 range, followed by the 20–50 range, while only six papers include more than 50 subjects. The bar plot on the right summarizes the number of studies per year, highlighting that most were published between 2022 and 2025, with fewer published during or before 2021. Another observation derived from Figure 6 concerns the evolution of dataset sizes over the years. Diagnosis, assessment, FoG detection, tremor, and gait analysis studies do not show a clear increasing trend. However, differential diagnosis and symptom quantification studies appear to have progressively increased cohort size over time, with the aim of achieving more generalizable models and results.

Another important aspect concerns the number of PD patients involved in each study and the distribution of disease severity levels in order to understand whether the disease is comprehensively represented and whether models can adequately identify it. To compute this statistic, we excluded studies involving only PD patients (thus having 100% PD in their dataset) and then computed the mean proportion of Parkinson’s subjects in the remaining acquisition protocols. The resulting average proportion of PD patients with respect to other subjects (e.g., ET or healthy controls) is 55.71±14.01%. This suggests that, on average, the distribution of Parkinson’s patients within datasets is relatively balanced. In some cases, the distribution is perfectly equal (50% in [62,63,85]); in other cases, it is skewed towards other subject groups (≈29% of PD in [69,70]); and in others it is strongly skewed towards PD (>70% of PD patients in [78,82,84]). This variability in class distribution may influence the training and evaluation of ML models. Strongly skewed datasets can lead to biased classifiers that favor the majority class, potentially inflating performance metrics such as accuracy while reducing the ability to correctly identify underrepresented subjects. Conversely, more balanced datasets allow models to learn more representative decision boundaries between PD and non-PD conditions. Furthermore, variability in PD proportions across datasets reflects differences in study design and clinical objectives. Some works focus primarily on characterizing Parkinson’s disease itself, which explains the high prevalence of PD patients, whereas others aim at differential diagnosis (e.g., PD vs. ET or PD vs. HC), resulting in more balanced or even PD-underrepresented distributions. This heterogeneity highlights the need for careful interpretation of reported model performance since results obtained on datasets with different class compositions may not be directly comparable. In particular, models trained on strongly PD-dominant datasets may show limited generalization when applied to real-world scenarios, where the prevalence of Parkinson’s disease is typically lower.

**Figure 7:**
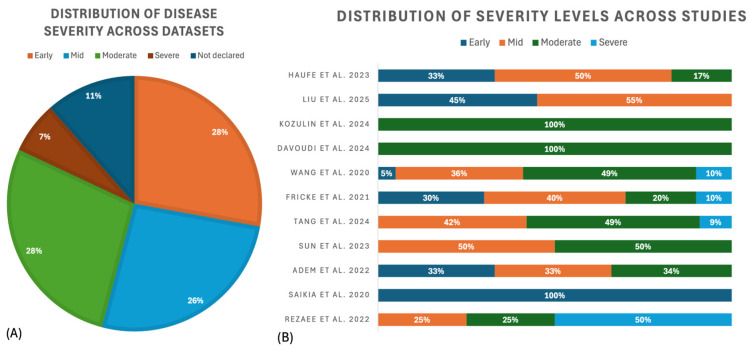
Considering a mapping of UPDRS and H&Y levels as 1 = early, 2 = mid, 3 = moderate, and 4 = severe, (**A**) shows the distribution of Parkinson’s disease severity levels across datasets. Each slice represents how many datasets included that severity level. Papers that did not declare patient severity are also included; (**B**) provides a more detailed view of how well each severity level is represented in the studies that explicitly reported the number of patients per score (Haufe et al. [87], Liu et al. [86], Kozulin et al. [83], Davoudi et al. [77], Wang et al. [76], Fricke et al. [70], Tang et al. [67], Sun et al. [66], Adem et al. [64], Saikia et al. [63], Rezaee et al. [62]).

Furthermore, Figure 7 reports the distribution of Parkinson’s disease severity levels across datasets. This further contributes to understanding dataset characteristics in relation to RQ2. In particular, we considered a typical mapping of levels as 1 = early stage, 2 = mid stage, 3 = moderate severity, 4 = severe state, and summarized the information provided by each study on the involved patients. Some works, however, did not specify the disease severity of their participants, and these were included under the label “Not declared”. Figure 7A already highlights a recurring limitation of Parkinson’s experiments, which is the lack of evaluation of more severe cases, due both to the clinical condition of affected patients and difficulty in involving them in experimental protocols. This limits the ability of trained models to correctly identify sEMG patterns associated with the full spectrum of PD severity. Additionally, to better understand the severity distribution among the studies that declared both severity level and number of patients per level, we generated a stacked bar plot (Figure 7B) showing how well each severity score is represented. Again, this further supports the conclusion that severe stages are underrepresented, potentially affecting model training and testing on unseen data.

Finally, to conclude the discussion on dataset variability, we examined whether the data used in each experiment are publicly available, available upon request, or completely private. Table 27 summarizes the availability of the datasets used in the reviewed studies. The majority of works rely on private datasets. Only a limited number of studies (two) provide fully public datasets, mainly in the context of FoG detection and bradykinesia assessment, while several datasets are available only upon request to the authors. This limited access represents an important challenge for the research community as it hinders reproducibility and fair comparisons among ML approaches. The lack of openly available datasets also limits the development of more robust and generalizable models for the analysis of Parkinson’s. It is also worth mentioning that, although not directly associated with any of the reviewed studies, a recent open-source dataset named ParkinSenseDB [100] has been released on the Mendeley Data repository. This dataset, reported in the last row of Table 27, contains multimodal recordings collected from 53 subjects (29 PD, 24 HC) using an optoelectronic system and wearable sensors, including sEMG and IMU signals acquired during gait tests. The availability of such data is particularly valuable for the development and benchmarking of machine learning approaches aimed at analyzing motor symptoms in Parkinson’s disease as it enables the investigation of both kinematic and muscular activity patterns. Resources such as ParkinSenseDB therefore represent an important step toward improving dataset availability and reproducibility in Parkinson’s disease research.

Overall, these observations highlight several limitations of the current datasets used in sEMG-based Parkinson’s disease studies, including limited cohort sizes, heterogeneous class distributions, and restricted data accessibility. This partially addresses RQ5, which tries to identify the main limitations and open challenges in sEMG research for Parkinson’s disease. Therefore, these aspects should be carefully considered when interpreting the reported model performance.

In addition to dataset characteristics, acquisition protocols represent another key factor in addressing RQ2 as they also exhibit substantial variability among the reviewed articles. All the studies relied on wearable sensors for data collection. The most common modalities include inertial measurement units (IMUs), sEMG, force sensors and pressure insoles, motion capture systems, electroencephalography (EEG), and skin conductance (SC) sensors. As the main focus of this review, all the selected works involve surface electromyography. Several studies employed commercial wearable platforms, such as Shimmer, Delsys Trigno, Myo Armband, or Dantec Keypoint systems, while others relied on custom-built acquisition setups. Differences in acquisition hardware may introduce intrinsic preprocessing characteristics (e.g., AC coupling effects acting as high-pass filters or built-in analog filtering stages) [41,42], which are often not explicitly reported. In addition, sampling frequencies vary considerably across studies and, for sEMG signals, are generally higher than for IMUs, typically ranging from 200 to 2 kHz, with some systems reaching up to 12 kHz. These factors can significantly influence the recorded signal and further limit the comparability and reproducibility of results. The adoption of more standardized acquisition protocols would therefore contribute to improving consistency across studies. IMUs are frequently used, particularly for tremor and gait analysis. For example, in the differential diagnosis category, all the studies adopted a multimodal configuration combining sEMG and accelerometer data. EEG was used in a limited number of works, mainly for diagnosis and FoG detection. Skin conductance and foot pressure sensors were also employed in specific applications, such as FoG detection and gait disorder classification. Among the 29 selected articles, 13 employed a unimodal acquisition based exclusively on sEMG, six combined sEMG and IMU, while the remaining 10 adopted a multimodal configuration integrating sEMG, IMU and additional modalities (e.g., biometric data, EEG, and SC). For tremor detection, almost all the studies except one employed a unimodal configuration, achieving their performance without additional modalities.

**Figure 8:**
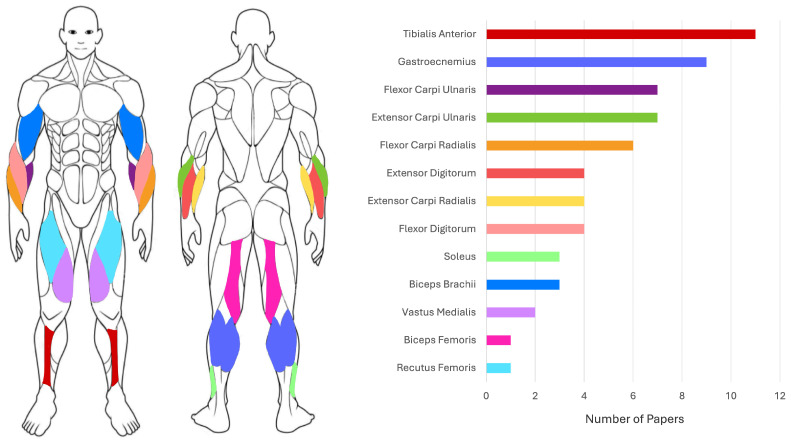
Distribution of the muscles monitored using sEMG in the reviewed studies. The body diagram highlights the anatomical locations of the recorded muscles, while the bar chart reports the number of studies in which each muscle was monitored.

To answer RQ2 it is also important to observe that sensor positioning varies depending on the targeted motor symptom, with wrist-mounted devices commonly used for tremor monitoring and lower-limb placements adopted for gait and FoG analysis. In order to correctly place the sEMG electrodes, some studies followed the Surface EMG for Non-Invasive Assessment of Muscles (SENIAM) guidelines, which help to standardize the acquisition protocol and improve comparability across datasets. However, only five works explicitly declared adherence to SENIAM, indicating that a standardized acquisition process is still lacking. Figure 8 shows the distribution of muscles monitored through sEMG. The muscles most frequently analyzed are the tibialis anterioris and gastrocnemius, which are commonly involved in gait and postural control and are therefore widely used in studies focusing on gait analysis and FoG detection. Upper-limb muscles such as flexor and extensor carpi ulnaris and radialis are also frequently monitored, particularly in studies investigating tremor and other upper-limb motor symptoms. Overall, the figure indicates that muscle selection strongly depends on the targeted motor task, with lower-limb muscles predominantly used for gait analysis and upper-limb muscles of the forearm and the wrist used for tremor assessment. At the same time, the variability in monitored muscles reflects the lack of a fully standardized acquisition protocol for sEMG recordings in Parkinson’s disease research. This heterogeneity further complicates comparison across studies and emphasizes the need for more standardized acquisition protocols.

Overall, the reviewed studies reveal that sEMG-based Parkinson’s disease research is characterized by heterogeneous datasets, variable acquisition protocols with different sensors and placements considered, and limited data accessibility, all of which impact reproducibility and model generalizability and replicability, thus providing a comprehensive answer to RQ2. To summarize, future studies would benefit from the creation of larger multi-center publicly available datasets and from the adoption of standardized acquisition protocols, which would facilitate reproducibility and enable fair comparison between machine learning approaches.

### 5.2. Signal Processing and Feature Engineering

After data acquisition and dataset organization, the next steps in the development of sEMG-based machine learning models involve signal preprocessing and feature engineering. This section addresses RQ3, aiming to identify the most common preprocessing techniques, feature extraction methods, and the most informative signal characteristics. Preprocessing aims to improve signal quality and reduce noise, thereby enabling the extraction of meaningful features that better describe the physiological characteristics of the signal. sEMG signals exhibit complex behavior and are typically noisy, non-stationary, and non-linear, making appropriate preprocessing necessary to improve signal quality and readability. Proper signal processing enables the extraction of relevant physiological information on PD from sEMG recordings. This is achieved during the feature engineering procedure, in which relevant characteristics are extracted and subsequently processed by the implemented models. Most studies follow a traditional pipeline based on manual feature extraction. The resulting feature map is then used as input to a shallow ML model for the classification or prediction of PD symptoms and for automatic severity assessment. However, others rely on automatic feature extraction, which is performed using deep learning models.

Regarding preprocessing, we aim to identify the most commonly adopted and reliable techniques for effective sEMG signal processing. In Table 28 we list the main techniques employed, together with their purpose and the number of studies adopting them. The main preprocessing techniques include filtering, rectification, smoothing, envelope extraction, resampling, normalization, and signal segmentation. Filtering is almost universally applied, although with some differences in implementation: some works directly bandpass filter in the sEMG range of interest, whereas others apply separate low-pass and high-pass filters for better control over the filtered spectrum. The typical sEMG frequency range is approximately (10–500) Hz, although the most commonly adopted range is 20–400/450 Hz. Furthermore, a common choice is to remove power-line interference using a notch filter, centered at 50 Hz for European acquisitions and 60 Hz for American acquisitions. Resampling may be required after filtering, and it is mainly applied in studies employing multimodal acquisitions, where standardization across sensor characteristics is necessary. Envelope extraction, often following smoothing and rectification, is less frequently performed, although it is useful for extracting muscle activation patterns, which may support both feature engineering and model training by improving the representation of relationships among signal characteristics. Normalization is typically applied as one of the final preprocessing steps because it reduces inter-subject variability and improves comparability across studies. The main techniques are amplitude normalization, Z-score normalization, and min–max scaling. Normalization based on maximum voluntary contraction (%MVC), which is the standard approach, is only applied in a limited number of studies, reflecting the practical difficulty of collecting such data during acquisition. Finally, another important and widely adopted step is segmentation, which divides the signal into overlapping windows to better represent its temporal evolution and alterations over time. This is mainly performed using sliding windows and requires selecting both the window length and the stride (i.e., overlap percentage). The identification of optimal segmentation parameters in sEMG analysis remains an open topic; however, the typical window lengths in the reviewed studies range from 250 ms to several seconds, with overlap values typically ranging from 0% to 50%. It is also interesting to analyze segmentation choices across different tasks. For diagnosis and assessment, only two studies [62,65] performed signal segmentation, both using a 500 ms window length with 30% and 50% overlap. For differential diagnosis, no study implemented segmentation, whereas, for FoG prediction, all five studies segmented their signals using a 3 s window length, with overlaps of 0%, 10%, or 90%. Two of these [71,72] also described how data labeling was handled after windowing through a PFG-based method with an optimally selected threshold, while [74] used a majority-voting strategy. In addition, all the tremor classification studies performed segmentation; however, the window lengths and overlap choices varied consistently and do not allow the identification of a standard configuration. A trade-off between temporal resolution and robustness therefore remains necessary.

Overall, the preprocessing pipelines are relatively consistent across studies. However, parameter choices vary both in relation to the specific study and the task addressed. This limits the possibility of identifying a common strategy for optimal sEMG signal processing. Moreover, this variability makes it difficult to directly compare and reproduce results, specifically when studies do not provide sufficient methodological details.

Regarding the feature extraction step, in general, two main paradigms are adopted for feature engineering: handcrafted manually designed features and automatic feature extraction through deep learning techniques. In this review, with the exception of two works that did not apply feature extraction because of the type of implementation performed (ref. [77] applied a clustering algorithm, whereas ref. [85] performed a statistical analysis), 55% of the articles used manual feature extraction, 26% exploited deep learning models for automatic extraction, and 19% used both approaches. Among the latter, the rationale for combining both techniques was either to enlarge the dataset, compare ML and DL performance, or extract information from one modality and fuse it with features extracted manually from the other modalities.

Handcrafted features are typically computed from different domains: time, frequency, power, and time–frequency (wavelet), with the addition of non-linear features that better capture the intrinsic nature of the sEMG signal. Table 29 reports these feature domains, their physiological meaning, examples of commonly computed features, and the number of studies that extracted features from each domain. Time-domain features are used in almost all the studies; they are the most commonly selected descriptors because they are computationally simple and effective for characterizing sEMG amplitude. Frequency- and power-domain features are also frequently used and are particularly relevant in PD studies as tremor-related signals often show distinct spectral peaks that help to characterize oscillatory muscle activation patterns. Time–frequency features, usually derived from wavelet transforms, and non-linear features are also useful for describing non-stationary and non-linear signals such as sEMG. However, these features were less commonly adopted in the reviewed studies.

**Figure 9:**
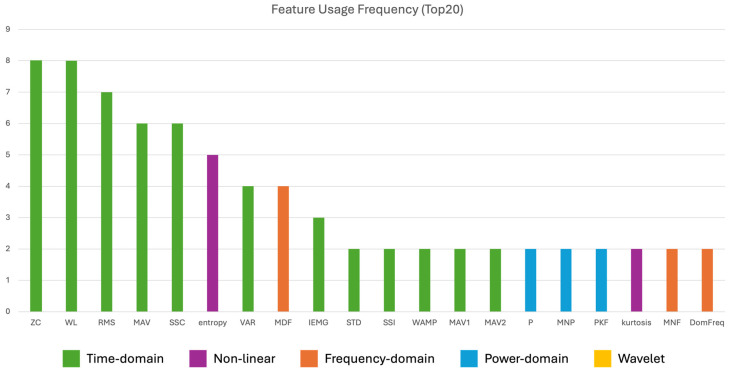
Frequency of adoption of sEMG features across the reviewed studies. Features are grouped by domain (time, frequency, power, time–frequency, and non-linear) and colored accordingly. Only features appearing in more than one study are shown, ordered from most to least frequently used.

Figure 9 shows the frequency of adoption of specific features across different domains. For better visualization, we limited the graph to features appearing in more than one study, resulting in a set of the 20 most frequently used features. This information may be valuable for future studies investigating the use of sEMG signals for training machine learning models as it highlights the most commonly used and representative features in sEMG-based PD evaluation and assessment.

At the end of manual feature extraction, the majority of the papers performed standardization or normalization in order to obtain a feature distribution that would not bias model performance.

As mentioned previously, some studies rely on automatic extraction through deep learning architectures. This choice typically enables the use of raw sEMG signals as model input, with the model returning a feature vector as output. Common architectures include CNNs, LSTMs, FCNs, and MLPs, often combined in hybrid configurations to exploit both spatial and temporal feature extraction. In some cases, cross-attention mechanisms are also used to fuse features extracted from different modalities or architectures. These techniques are potentially more powerful because they can identify patterns and relationships directly within the sEMG signal; however, they require larger datasets to perform adequately and avoid overfitting. This explains why automatic feature extraction is still less commonly employed in this research field: small datasets limit the effectiveness of DL techniques.

A total of 12 studies also applied dimensionality reduction or feature selection. Among the most adopted techniques are principal component analysis (PCA), statistical importance analysis (e.g., paired *t*-tests), and the Relief-F algorithm for handcrafted features; and attention mechanisms, ensemble methods (e.g., soft voting), and architectural optimizations (e.g., ResNet) for automatic feature extraction using DL algorithms. Their objectives are, respectively, to identify the signal characteristics containing the most relevant information and to reduce the dimensionality of the feature maps used as input to DL models. This helps to minimize overfitting while reducing computational and memory requirements. The most informative sEMG features generally correspond to those shown in Figure 9. In particular, time-domain features such as RMS, MAV and WL are among the most representative descriptors of muscle activation amplitude and signal complexity; frequency-domain features such as mean frequency, median frequency, and spectral power are useful for interpreting tremor-related oscillatory movements; non-linear features, such as entropy (sampEn, permEn, and apEn) reflect changes in signal complexity due to neuromuscular impairment.

Overall, these findings indicate that preprocessing pipelines are relatively consistent but not fully standardized. This limited adoption of common signal processing steps hinders reproducibility and comparability across studies. Feature engineering in sEMG-based Parkinson’s disease studies predominantly relies on handcrafted time- and frequency-domain descriptors, which remain the most informative and widely adopted signal characteristics. This is due to the limited size of the available datasets, which constrains the use of DL approaches. Furthermore, feature selection algorithms should be explored further to identify a common set of sEMG features to represent PD characteristics. These conclusions provide a comprehensive answer to RQ3.

### 5.3. Machine Learning and Deep Learning Performance

The final major step in sEMG-based Parkinson’s research is model development for classification or prediction of specific disease characteristics. The reviewed studies can be grouped into two main paradigms: traditional machine learning and deep learning model evaluation. Some studies employed shallow models, such as support vector machines, k-nearest neighbors, and random forests, while others explored more advanced techniques, such as convolutional neural networks, multilayer perceptrons, and long short-term memory architectures. These are generally more complex and require larger datasets but can automatically learn relevant representations of PD-related patterns from the sEMG signal without relying on handcrafted features. This section addresses RQ4, aiming to identify the most frequently adopted models and to compare their performance across different tasks. The aim is also to determine whether there is a best pipeline for model choice, parameter configuration, and training strategy.

**Figure 10:**
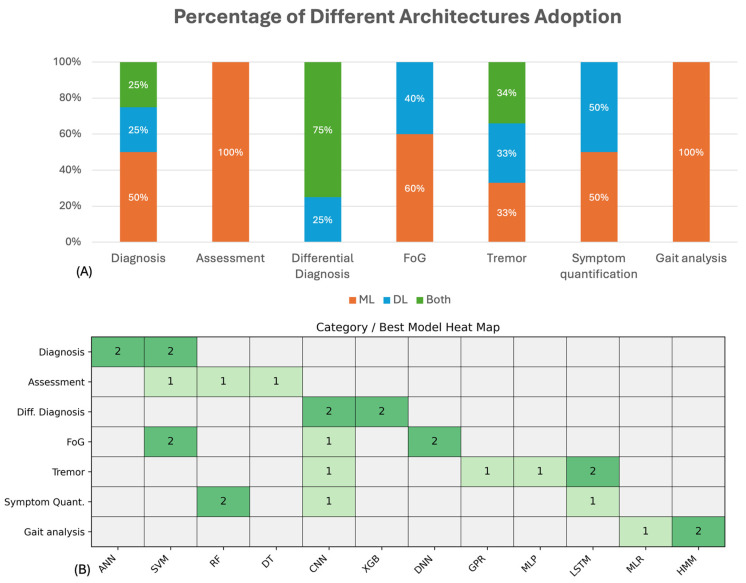
Adoption of machine learning and deep learning architectures across Parkinson’s disease analysis tasks. (**A**) Percentage of studies employing traditional ML, DL, or both approaches across the considered application categories. (**B**) Heatmap showing the models reported as best-performing in each category. The numbers indicate how many studies identified a specific model as achieving the highest performance for that task.

We compared studies employing traditional machine learning models with those adopting deep learning architectures, as well as those evaluating both paradigms, to identify the best configuration. Figure 10 provides an overview of these trends. From Figure 10A it is possible to quantify the proportion of papers that focus on each of these approaches within each category. In general, 54% of the studies employed only traditional ML models, with the most commonly adopted being SVM, RF, LR, XGBoost, and DT. Furthermore, 24% of the works focused exclusively on deep learning architectures, including CNN, LSTM, MLP, hybrid models (e.g., CNN + LSTM), and attention-based architectures. The remaining 22% evaluated both paradigms to compare their performance and identify the most suitable approach for the specific task. An interesting observation from Figure 10A is that, for disease assessment and gait analysis tasks, the reviewed studies were exclusively based on shallow ML algorithms. This suggests that further investigation of DL approaches in these areas may be beneficial. Figure 10B provides additional insight by showing the models identified as the best-performing within each category. For example, the first row of the heatmap indicates that, in the diagnosis task, two studies achieved the best results using ANN, while two others reported SVM as the most effective model to distinguish PD patients from healthy subjects. Overall, the models most frequently reported as best-performing are SVM and CNN (18% each), followed by LSTM (11%). Across categories, some patterns can be observed: in PD diagnosis, ANN and SVM appear to be particularly effective for distinguishing PD patients from healthy subjects; in differential diagnosis, CNN and XGBoost show strong performance in identifying different neurological conditions; while, in FoG prediction, SVM performs well among traditional ML approaches, with CNN- and DNN-based models also achieving competitive results.

To better understand the factors influencing model performance, it is also important to evaluate training and validation strategies. In biomedical applications, it is essential to ensure that the model generalizes well across different subjects. The reviewed studies mainly adopted three types of validation methods: standard train–test splits based on a selected train–test split percentage, k-fold cross-validation (with different values of k), and leave-one-subject-out cross-validation (LOSO). Table 30 shows how many studies adopted these strategies. Among those explicitly reporting the technique used, 19% used a standard split validation strategy, 48% applied a k-fold CV, typically with 5 or 10 folds, and the remaining 33% applied LOSO, in which the model is trained on the data of all the subjects except one, which is used for testing. This procedure is repeated for all subjects, which allows evaluation of model generalization on unseen individuals. LOSO is particularly suitable for physiological datasets. Compared with k-fold CV, LOSO provides a more realistic assessment of model performance in real-world clinical scenarios, where models must generalize to unseen patients. Nevertheless, some studies still relied on random train–test splits or standard k-fold cross-validation, which can lead to optimistic performance estimates if samples from the same subject appear in both training and testing sets, thus introducing data leakage.

**Figure 11:**
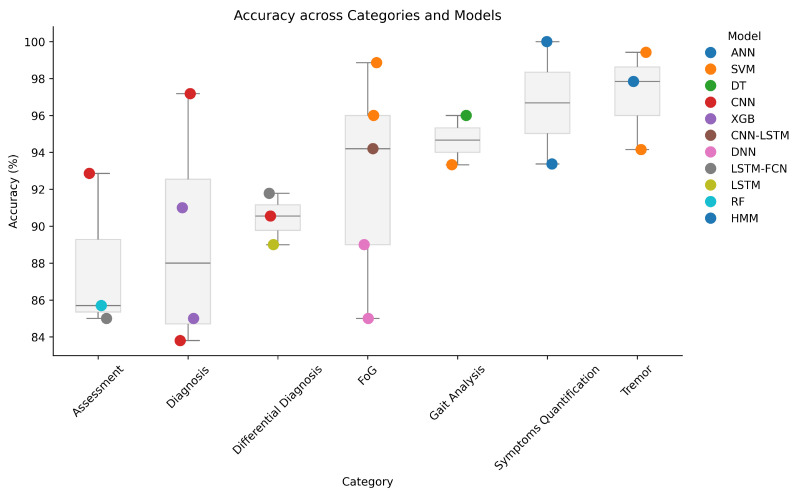
Accuracy achieved by the best-performing models reported in the literature for different sEMG-based Parkinson’s disease analysis tasks. Each point represents a study and is color-coded according to model type. Box plots summarize the distribution of accuracies within each category, enabling comparison of performance variability across tasks.

For a better comparison of the best-performing models across categories, Figure 11 summarizes the classification accuracies reported by the various studies. To allow a fair comparison, we excluded from this evaluation all papers that did not report accuracy as a metric ([63,65,76,78,83,87]). Different colors represent the model achieving that performance, while the points represent individual studies grouped by category. In addition, the box plots provide a visual summary of accuracies within each category, highlighting variability and central tendency. From this figure, some differences in performance across categories can be observed. Tasks related to tremor detection and symptom quantification generally achieve the highest accuracy, often exceeding 95%. These tasks typically involve more distinctive sEMG patterns, which may facilitate classification. Similarly, FoG detection and gait analysis show relatively high median accuracies, although with slightly greater variability across studies. Conversely, tasks such as diagnosis and assessment exhibit a wider spread of results and lower median accuracies. This variability may be attributed to the higher complexity of these tasks, which often require distinguishing subtler neuromuscular alterations, or to heterogeneous datasets and experimental protocols. To complement the detailed comparison provided by Figure 11, Table 31 offers a more general overview of the comparison between machine learning and deep learning approaches. Specifically, it reports the performance achieved by the best-performing model for each study, considering only those that provide a measure of classification accuracy. While the figure emphasizes the performance variability within each task, the table provides a clearer view of which approach achieves the highest performance across studies. Regarding model types, as discussed above, both traditional ML approaches (such as SVM and decision trees) and DL architectures (e.g., CNN-based models) appear among the best-performing methods across several categories. However, no single model or specific approach consistently dominates, suggesting that model effectiveness strongly depends on the specific application and dataset characteristics.

Comparing model performance across studies remains challenging due to the lack of standardized evaluation metrics. Although multiple performance measures are reported in the literature depending on the task, including precision, recall, F1-score, area under the ROC curve (AUC), or regression metrics such as mean absolute error (MAE) and root mean squared error (RMSE), classification accuracy remains the most commonly reported metric and was therefore selected for comparative purposes in this review. However, it should be highlighted that accuracy alone is not sufficient to fully characterize model performance, particularly for imbalanced classification tasks and heterogeneous datasets. Metrics such as sensitivity, specificity, and balanced accuracy would provide a more comprehensive evaluation; therefore, the reported comparisons should be interpreted with caution. Moreover, for severely imbalanced datasets, even sensitivity and specificity can be misleading in isolation [103], and metrics such as F1-score, Matthews Correlation Coefficient (MCC), or G-Mean should be considered to provide a more robust evaluation [104]. Moreover, the relatively small size of many datasets may lead to inflated performance estimates, particularly when validation strategies do not properly separate subjects between training and testing. In fact, as partially discussed in Section 5.1, dataset size is an important factor influencing model performance. Several studies relying on traditional ML approaches report competitive or superior performance compared with DL models, particularly when datasets are small. This suggests that the effectiveness of deep learning architectures in sEMG-based analysis may currently be limited by the availability of sufficiently large and diverse datasets.

**Figure 12:**
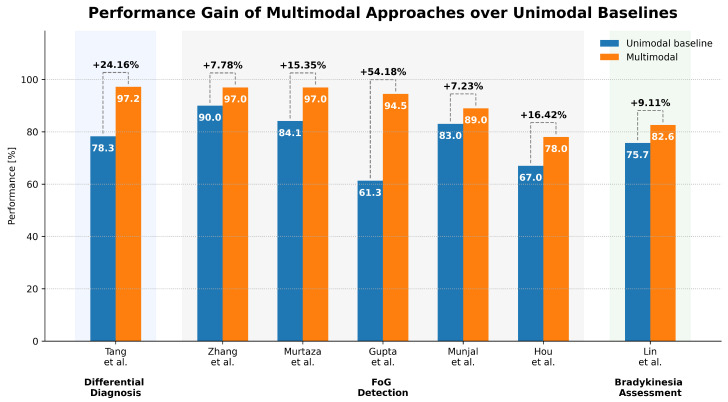
Comparison between unimodal and multimodal approaches in studies explicitly evaluating both configurations (Tang et al. [67], Zhang et al. [71], Murtaza et al. [72], Gupta et al. [73], Munjal et al. [74], Hou et al. [75], Lin et al. [82]). Bars represent reported performance values, while dashed annotations indicate relative improvements from multimodal fusion. Studies are grouped by task. Unimodal values correspond to the worst-performing single modality reported in each study.

It is also important to examine whether combining sEMG signals with other sensing modalities improves model performance. In this context, multimodal approaches integrating sEMG with IMUs, accelerometers, or EEG signals have been increasingly explored. Across the reviewed literature, seven studies explicitly compared unimodal and multimodal configurations, consistently reporting improved performance when multiple modalities were considered. The observed performance gains vary depending on the task and dataset but generally range from approximately 6% to over 30%. For instance, in differential diagnosis, Tang et al. [67] reported an improvement of about 14.5% when combining sEMG and accelerometer data compared with single-modality inputs. Similarly, in FoG prediction, the performance increases ranged from approximately 6% (Zhang et al. and Munjal et al. [71,74]) to 31.8% (Gupta et al. [73]) across different studies, while, in bradykinesia assessment, Lin et al. [82] observed an improvement of about 6.9% These results are summarized in Figure 12, which highlights the performance gains achieved by multimodal approaches across different tasks. For unimodal performance, the worst-performing single modality reported in each study (e.g., “sEMG only” and “ACC only”) was selected for comparison. The largest improvements are observed in FoG prediction tasks, where all the relevant studies evaluated multimodal configurations, suggesting that sensor fusion is particularly beneficial for complex motor patterns involving both muscular and kinematic components. This trend can be explained by the complementary nature of the acquired signals: sEMG provides information on muscle activation patterns, whereas inertial sensors capture the kinematic characteristics of movement. Their combination enables models to build a more comprehensive representation of Parkinson’s disease motor symptoms, improving both robustness and classification performance. Consistent findings have also been reported in the broader wearable sensing literature. For example, Biswas et al. [105] demonstrated that sensor fusion-based activity recognition models for patients’ daily life monitoring achieve higher performance compared to single-sensor approaches. Similarly, Celik et al. [106] showed that combining sEMG and IMU sensors improves gait analysis in PD patients because the joint measurement of muscle activation and kinematic features enables a more accurate representation of gait patterns. Furthermore, for upper-limb movement prediction, multimodal frameworks provide higher fidelity in capturing kinematic dynamics compared to single-modality approaches, as reported by Fritsche et al. [107]. Overall, while sEMG alone provides valuable insights into neuromuscular activity, the current evidence suggests that it is generally more effective when integrated within a multimodal framework, particularly in complex clinical tasks requiring comprehensive symptom characterization. However, the reported improvements are not always directly comparable due to differences in datasets, acquisition protocols, and evaluation strategies and should therefore be treated as indicative rather than conclusive.

In conclusion, although many studies report high classification performance, these results should be interpreted with reservation. A large proportion of the studies in the reviewed literature rely on small datasets with fewer than 50 subjects, which increases the risk of overfitting and limits model generalizability. In such settings, complex models such as DL architectures may capture dataset-specific patterns rather than disease-related characteristics, hindering their applicability to unseen data. Moreover, the use of validation strategies that do not strictly separate subject-specific data between training and testing sets (e.g., random split or k-fold CV) may lead to overly optimistic performance due to data leakage. While leave-one-subject-out CV provides a more realistic evaluation, it is not consistently adopted among the studies. From a cross-study perspective it is also important to note that no clear and consistent evidence supports the superiority of DL approaches over traditional ML models when evaluated under comparable conditions. The reported performance differences are influenced by the number of samples, feature representation and validation strategy rather than by the model itself. Similarly, studies incorporating multimodal data acquisition generally report improved performance compared to unimodal sEMG-based approaches, highlighting the value of combining complementary physiological signals. However, these gains are often observed on small and task-specific datasets, and their robustness across larger populations and real-world conditions for PD remains to be validated. In addition, although the preprocessing pipelines are similar, the impact of specific parameter choices (e.g., filtering ranges, segmentation strategies, and normalization techniques) is rarely evaluated, limiting the identification of optimal and reproducible configurations. These methodological limitations, combined with the heterogeneity of acquisition protocols and signal processing pipelines, make it difficult to assess whether the reported performance can generalize to independent datasets or real-world clinical scenarios. Therefore, the current results should be viewed as promising but preliminary, highlighting the need for larger standardized datasets and more rigorous validation frameworks.

### 5.4. Limitations and Future Research Directions

Despite the identified promising results, several limitations currently hinder the development of robust and clinically applicable machine learning systems based on sEMG signals for the analysis of Parkinson’s disease. This section addresses RQ5, focusing on the main limitations and open challenges in this field.

One of the most evident limitations concerns the relatively small size of the datasets. As highlighted in previous sections, most studies rely on cohorts containing fewer than 50 subjects. Although such datasets may be sufficient to explore preliminary methodological approaches, they limit the generalizability of the developed models. In particular, deep learning architectures typically require larger and more diverse datasets to fully exploit their potential without overfitting.

In addition, the limited availability of publicly available datasets represents a significant obstacle to reproducibility and benchmarking. Most of the reviewed studies rely on non-public datasets or data available upon request, which prevents independent validation of the proposed approaches and limits fair comparison among different machine learning models.

Another important challenge is the heterogeneity of acquisition protocols and signal processing pipelines. Differences in electrode placement, monitored muscles, sampling frequencies, and preprocessing techniques make it difficult to directly compare results across studies. Although some works adopt guidelines such as SENIAM for electrode placement, a standardized acquisition protocol for EMG-based Parkinson’s disease analysis is still lacking.

A further limitation is related to variability in the evaluation metrics and validation strategies adopted across studies. Although leave-one-subject-out cross-validation is often used to ensure realistic model evaluation in biomedical contexts, other studies rely on random train–test splits or k-fold cross-validation, which may lead to optimistic performance estimates when samples from the same subject appear in both training and testing sets. Furthermore, the use of different performance metrics complicates direct comparison between models developed for similar tasks.

Based on these limitations, several key directions for future research can be identified. First, the creation of larger, more diverse, and publicly available benchmark datasets would support the development of more robust and generalizable machine learning models while enabling fair comparison among algorithms and accelerating methodological progress in the field. In this context, the exploration of data balancing and augmentation techniques represents a promising strategy to mitigate the limitations associated with small and imbalanced datasets. These approaches can enhance model robustness, reduce overfitting, and improve generalization to unseen data, particularly in biomedical applications where data collection is inherently challenging. Recent work in the broader wearable sensing domain has demonstrated the effectiveness of such strategies. For example, Trabassi et al. [108] showed the effectiveness of data balancing techniques and generative AI algorithms in improving classification performance in rare neurological disease datasets characterized by limited and skewed samples, particularly in the analysis of gait abnormalities. Similarly, Sabbatini et al. [109] explored different imbalanced data management techniques in machine learning models for sleep stage classification using non-invasive sensor modalities, showing that these strategies can improve model performance. Although these studies focus on other healthcare domains, the underlying principles are directly applicable to sEMG-based Parkinson’s disease research, where similar challenges related to dataset size and skewness have been identified and are frequently observed. However, only limited evidence of their adoption was found in the reviewed sEMG-based PD studies, highlighting an important opportunity for future research in this field. Second, the adoption of standardized acquisition protocols and signal processing pipelines would improve comparability across studies and facilitate reproducibility. Finally, future studies should further explore the potential of multimodal approaches, combining sEMG signals with complementary sensing modalities such as inertial measurement units, accelerometers, or electroencephalography. Such approaches could provide a more comprehensive representation of motor symptoms and potentially improve the accuracy and robustness of Parkinson’s disease assessment systems.

### 5.5. Real-World Implementation of sEMG in Parkinson’s Disease

While numerous studies have demonstrated the potential of surface electromyography (sEMG) for the quantitative assessment of Parkinsonian motor symptoms, its translation into real-world clinical practice is essential for maximizing its clinical impact. Advances in wearable and wireless technologies have enabled the development of portable sEMG systems that are capable of unobtrusive monitoring in both clinical and home environments. These systems facilitate valid long-term data acquisition, allowing the assessment of motor symptoms during activities of daily living.

Continuous monitoring through wearable sEMG provides objective and quantitative information that complements traditional clinical rating scales, such as the MDS-UPDRS, which are inherently limited to episodic and subjective evaluations. Longitudinal recordings can capture motor fluctuations related to medication cycles, disease progression, and treatment response. Beyond tremor, sEMG has been applied to the assessment of bradykinesia, rigidity, gait-related muscle activation patterns, and freezing of gait, thereby offering a comprehensive characterization of motor dysfunction in PD.

The integration of sEMG-derived metrics into clinical workflows represents a key step toward precision medicine in Parkinson’s disease. Automated signal processing and machine learning techniques allow the extraction of clinically relevant features, such as muscle activation timing, amplitude, frequency characteristics, and co-contraction indices. These measures can support clinical decision-making, including therapy optimization and treatment evaluation (e.g., fine-tuning deep brain stimulation parameters).

From a rehabilitation perspective, sEMG can provide valuable insights into neuromuscular control and compensatory strategies, supporting the development of personalized physiotherapy programs and enabling objective monitoring of treatment outcomes in order to adapt rehabilitation strategies accordingly.

Despite its advantages, several challenges limit the real-world implementation of sEMG. Electrode placement variability, sensitivity to artifacts (e.g., sweating and motion), and day-to-day as well as medication-related fluctuations can affect signal reliability and complicate longitudinal analysis, especially in unsupervised settings. In addition, usability and patient adherence remain critical issues as sEMG systems require more complex setup procedures compared to inertial measurement units (IMUs).

From a clinical perspective, the interpretability of sEMG-derived metrics is still limited as translating complex signal features into actionable insights is not always straightforward. While IMU-based systems are more widely adopted for real-world monitoring due to their robustness and ease of use, sEMG provides unique physiological information by directly capturing muscle activation patterns, enabling the assessment of neuromuscular control and coordination. Therefore, sEMG may offer added value in specific applications, such as the analysis of bradykinesia, rigidity, and rehabilitation outcomes, whereas IMU-only systems may be more suitable for continuous long-term monitoring. A multimodal approach combining sEMG and inertial sensors may represent a promising compromise.

To support future research and improve reproducibility, we propose a checklist of minimum reporting requirements for sEMG-based Parkinson’s disease studies (Table 32). This checklist summarizes the key methodological aspects across the full pipeline, including acquisition protocols, dataset characteristics, signal processing, feature engineering and model evaluation. The parameters reported in the table as Recommended/Common Practice correspond to either the best-performing or the most commonly adopted settings among the reviewed studies. The findings of this review should be viewed as a structured overview of the current trends and should be interpreted with caution due to the relatively limited number of available studies. This constraint may affect the generalizability of the findings and highlights the need for further large-scale standardized investigations.

Overall, the adoption of wearable, non-invasive, and low-power sEMG systems, combined with standardized methodologies, has the potential to bridge the gap between laboratory research and clinical practice, enabling continuous monitoring and more informed clinical decision-making.

## 6. Conclusions

This review provides a structured and comprehensive analysis of the recent research on the use of surface electromyography (sEMG) signals combined with machine learning and deep learning techniques for Parkinson’s disease assessment. The examined studies demonstrate that sEMG-based approaches can effectively support multiple clinical tasks, including diagnosis, symptom quantification, freezing of gait detection, and differential diagnosis. The analysis reveals several key trends. Traditional ML models remain the most widely adopted due to their robustness on limited datasets, while DL approaches are increasingly explored but still constrained by data availability. In addition, multimodal approaches generally show improved performance, suggesting that the integration of complementary sensing modalities is a promising direction. Despite encouraging results, the literature is characterized by significant heterogeneity in datasets, acquisition protocols, preprocessing pipelines, and evaluation methodologies. This lack of standardization limits reproducibility, comparability, and model generalization. Furthermore, only a limited number of studies address real-time implementation, interpretability, and integration with clinical workflows, which remain critical barriers to real-world adoption. Future research should therefore focus on the development of larger standardized datasets, the adoption of reproducible evaluation frameworks, and the design of multimodal systems that are capable of capturing the complexity of PD motor symptoms. Addressing these challenges will be essential to enable the translation of sEMG-based machine learning systems into reliable and clinically applicable tools. Ultimately, the integration of such approaches into clinical practice could support more objective, continuous, and personalized monitoring of Parkinson’s disease progression.

In summary, this review addressed the research questions as follows: (RQ1) sEMG is widely applied in PD research across multiple tasks, including diagnosis, symptom quantification, tremor analysis, freezing of gait detection, and differential diagnosis; (RQ2) the current studies rely on heterogeneous datasets and acquisition protocols, often characterized by limited cohort sizes and limited standardization, impacting reproducibility and model generalization; (RQ3) signal processing pipelines are relatively consistent across studies, with time- and frequency-domain features representing the most informative characteristics for sEMG-based PD analysis; (RQ4) no single ML or DL model significantly outperforms the others across tasks; both approaches achieve competitive performance depending on the dataset characteristics and application context; (RQ5) the key limitations include limited data availability, lack of standardized methodologies, and insufficient clinical validation, highlighting the need for more robust, reproducible, and clinically oriented research.

In this context, the authors’ future work will focus on addressing the identified limitations by investigating both ML and DL approaches for the analysis of sEMG signals acquired from PD patients. Particular attention will be given to the use of larger multimodal datasets to improve model robustness and performance. Furthermore, the proposed approaches will be evaluated across multiple datasets to assess their generalization capability and applicability to unseen data, supporting their potential use in real-world clinical scenarios.

## Figures and Tables

**Table 1:** Overview of the studies included in the review organized by application topic. For each paper, the reference, publication year, and the main objective of the proposed approach are reported.

Topic	Reference	Year	Scope
Diagnosis and Assessment	Loconsole et al. [61]	2018	PD vs. HC
	Rezaee et al. [62]	2022	PD vs. HC
	Saikia et al. [63]	2020	PD vs. HC
	Adem et al. [64]	2022	PD vs. HC and severity prediction
	Kleinholdermann et al. [65]	2021	UPDRS prediction
	Sun et al. [66]	2023	H&Y prediction
Differential Diagnosis	Tang et al. [67]	2024	PD vs. ET
	Xing et al. [68]	2022	PD vs. ET
	Sil et al. [69]	2025	PD vs. ET
	Fricke et al. [70]	2021	Gait disorders
FoG Prediction	Zhang et al. [71]	2022	Public dataset validation
	Murtaza et al. [72]	2025	Multimodal sensor features
	Gupta et al. [73]	2024	FoG early prediction
	Munjal et al. [74]	2025	TinyML evaluation
	Hou et al. [75]	2023	Deployment on edge
Tremor Analysis	Wang et al. [76]	2020	DBS efficacy evaluation
	Davoudi et al. [77]	2024	Biofeedback therapy evaluation
	Zanini et al. [78]	2019	Early prediction
	Lin et al. [79]	2023	Mild tremors for PD vs. HC
	Qin et al. [80]	2019	Severity quantification
	Farhani et al. [81]	2022	Type classification
Gait Analysis and Motor Symptoms	Lin et al. [82]	2025	Bradykinesia quantification
	Kozulin et al. [83]	2024	Bradykinesia quantification
	Guo et al. [84]	2019	Gait disorder classification
	Alves et al. [85]	2025	Rigidity quantification
	Liu et al. [86]	2025	Gait scoring (UPDRS)
	Haufe et al. [87]	2023	Gait event prediction
	Bengacemi et al. (2021) [88]	2021	Activity detection
	Bengacemi et al. (2024) [89]	2024	Activity detection + PD vs. HC

**Table 2:** Overview of collected datasets, used sensors, observed muscles and acquisition protocols for studies focusing on ML- and DL-based PD diagnosis using sEMG data.

Study	Subjects	Sensors	Muscles	Protocol
Loconsole et al. [61]	18 (11 PD, 7 HC)	Myo armband, WACOM tablet (sEMG + biometric)	Forearm	3 writing tasks
Rezaee et al. [62]	8 (4 PD UPDRS = 2, 3, 4, 4 HC)	PowerLab system (sEMG)	ECRB, FCU	Daily life (56 days)
Saikia et al. [63]	60 (30 PD H&Y = 1, 1.5, 30 HC)	Biophysical recorder (sEMG + EEG)	ECU, FDS	Wrist flexion/ extension
Adem et al. [64]	25 (15 PD, UPDRS = 2–4 10 HC)	SCU-7 sEMG	FCR, BB	Upper-limb movements

ECRB: extensor carpi radialis brevis; FCU: flexor carpi ulnaris; ECU: extensor carpi ulnaris; FDS: flexor digitorum superficialis; FCR: flexor carpi radialis; BB: biceps brachii.

**Table 3:** Comparison of preprocessing procedures, windowing strategy and choices, and additional signal handling techniques across studies analyzing sEMG signals with ML and DL techniques for a PD diagnosis.

Study	Preprocessing	Windowing	Other Techniques
Loconsole et al. [61]	Not declared	Not declared	-
Rezaee et al. [62]	High-pass (10 Hz), low-pass (500 Hz), notch (50 Hz)	500 ms length 30–50% overlapping	-
Saikia et al. [63]	Bandpass (5–500 Hz) MVC% normalization	No (30 min duration)	-
Adem et al. [64]	Notch (50 Hz), low-pass (500 Hz), high-pass (20 Hz)	No (5 s duration)	Raw data augmentation

**Table 4:** Feature extraction and feature selection techniques applied for each PD diagnosis-focused study. The last column summarizes the best sEMG features identified by each study either using a feature selection algorithm or not.

Study	Feature Extraction	Feature Selection	Best sEMG Features
Loconsole et al. [61]	Handcrafted	✓ (PCA)	RMS, ZC
Rezaee et al. [62]	Automatic	✓ (soft voting)	-
Saikia et al. [63]	Handcrafted	✗	P, RMS, WL, STD, MNF, MDF
Adem et al. [64]	Handcrafted	✓ (Relief-F)	En, MNF, skew, MDF, kurt, MFL, RMS, IEMG, MAV

✓: used a feature selection technique; ✗: did not use a feature selection technique; PCA: principal component analysis; RMS: root mean square; ZC: zero crossings; P: power; WL: waveform length; STD: standard deviation; MNF: mean frequency; MDF: median frequency; En: entropy; skew: skewness; kurt: kurtosis; MFL: maximum fractal length; IEMG: integral EMG; MAV: mean absolute value.

**Table 5:** Dataset splitting percentages, training method, evaluated models with selected hyperparameters declared or optimized and with which algorithm. The last column shows the performance reached by each study in terms of accuracy. When a study performed a comparison between more models, the one written in italic is the one reaching the highest performance.

Study	Train, Test, Val Split (%)	Training Method	Models	Hyperparameters	Performance
Loconsole et al. [61]	60, 20, 20	5-fold CV	*ANN*, *SVM*	MOGA optimization	Acc = 97.84%
Rezaee et al. [62]	60, 20, 20	5-fold CV	*SVM*–*RBF*	Default	Acc = 99.42%
Saikia et al. [63]	70, 15, 15	-	*Binary ANN*	Not declared	Not declared
Adem et al. [64]	80, 20, -	CV	*SVM*–*RBF*, kNN, LDA	Not declared	Acc ≈ 94.15%

**Table 6:** Dataset size, type of sensors used, muscles observed and acquisition protocols for the studies regarding sEMG-based PD severity assessment through ML and DL techniques.

Study	Subjects	Sensors	Muscles	Protocol
Adem et al. [64]	15 PD (5 early, 5 moderate, 5 advanced)	SCU-7 sEMG	FCR, BB	Upper-limb movements
Kleinholdermann et al. [65]	45 PD (UPDRS ON = 33±19.4, OFF = 45.7±23.1)	Myo armband (sEMG)	EDC, FDS	Tapping task (5 s)
Sun et al. [66]	28 PD (H&Y = 2, 3)	Delsys Trigno Avanti (sEMG)	GL, TA	Standing (20 s)

FCR: flexor carpi radialis; BB: biceps brachii; EDC: extensor digitorum communis; FDS: flexor digitorum superficialis; GL: gastrocnemius lateralis; TA: tibialis anterioris.

**Table 7:** Comparison of preprocessing procedures, windowing approaches, and additional signal handling techniques across sEMG-based PD severity assessment studies.

Study	Preprocessing	Windowing	Other Techniques
Adem et al. [64]	Notch (50 Hz), low-pass (500 Hz), high-pass (20 Hz)	No (5 s duration)	Raw data augmentation
Kleinholdermann et al. [65]	High-pass (10 Hz), adaptive notch (50 Hz)	500 ms length 250 ms step	-
Sun et al. [66]	Notch (50 Hz), detrending, amplitude norm., rect.	No (10 s duration)	-

**Table 8:** Feature extraction and feature selection methods for each study focused on Parkinson’s disease severity assessment using sEMG data and machine learning models.

Study	Feature Extraction	Feature Selection	Best sEMG Features
Adem et al. [64]	Handcrafted	✓ (Relief-F)	En, MNF, skew, MDF, kurt, MFL, RMS, IEMG, MAV
Kleinholdermann et al. [65]	Handcrafted	✗	(1) Hudgins’ (MAV, ZC, WL, SSC) [90] (2) Du’s (IAV, VAR, WL, ZC, SSC, WAMP) [91], (3) RMS
Sun et al. [66]	Handcrafted + signal (Fisher)	✗	RMS, kurt, %REC, sampEn, MPF, MDF

✓: used a feature selection technique; ✗: did not use a feature selection technique; En: entropy; MNF: mean frequency; skew: skewness; MDF: median frequency; kurt: kurtosis; MFL: maximum fractal length; RMS: root mean square; IEMG: integrated EMG; MAV: mean absolute value; ZC: zero crossing; WL: waveform length; SSC: slope sign change; IAV: integrated absolute value; VAR: variance; WAMP: Willison amplitude; %REC: recurrence rate; sampEn: sample entropy; MPF: mean power frequency.

**Table 9:** Dataset splitting, training method, models evaluated and performance reached for each study involved in PD evaluation. Where the study did a comparison between more models, the italic one in the column “Models” is the one that reaches the highest performance, shown in the last column.

Study	Train, Test, Val Split (%)	Training Method	Models	Hyperparameters	Performance
Adem et al. [64]	80, 20, -	CV	*SVM*–*RBF*, kNN, LDA	Not dec.	Acc ≈ 93.33%
Kleinholdermann et al. [65]	90, 10, -	10-fold CV	LR, SVM-poly, kNN, *RF*	Grid search	r = 0.853
Sun et al. [66]	Not declared	5-fold CV	LDA, SVM, kNN, *DT*	Not dec.	Acc = 96%

**Table 10:** Dataset and acquisition protocols for the studies analyzing the differential diagnosis task using the sEMG signal.

Study	Subjects	Sensors	Muscles	Protocol
Tang et al. [67]	140 (87 PD UPDRS = 2–4, 53 ET)	Dantec Keypoint (ACC + sEMG)	ECR, FCR	7 postures: rest, stretch (+1 kg), wing (+1 kg), vertical wing (+1 kg)
Xing et al. [68]	398 (257 PD, 141 ET)	Dantec Keypoint (ACC + sEMG)	flex. and ext. of forearm	4 postures: rest, stretch, wing, vertical wing
Sil et al. [69]	396 (124 PD UPDRS = 4.94 ±1.29, 272 ET)	Not declared (ACC + sEMG)	ECU, FCU	3 Postures: rest, posture (+1 kg)
Fricke et al. [70]	37 (18 dis. UPDRS = 1–7, 19 HC)	Noraxon system (ACC + sEMG)	RF, VM, BF, TA, GL	TUG + walk + turn + tandem gait

FCR: flexor carpi radialis; ECR: extensor carpi radialis; FCU: flexor carpi ulnaris; ECU: extensor carpi ulnaris; RF: rectus femoris; VM: vastus medialis; BF: biceps femoris; TA: tibialis anterioris; GL: gastrocnemius lateralis.

**Table 11:** Preprocessing and windowing choices for the studies that implemented ML or DL techniques for differential diagnosis using sEMG signals. In the last column, additional techniques that might have been used alongside the preprocessing step are reported.

Study	Preprocessing	Windowing	Other Techniques
Tang et al. [67]	Z-score	No (30 s duration)	ACC and EMG data recombination
Xing et al. [68]	ML: null -> mean, Z-score CNN: downsample, (2, 20) Hz filter, Z-score	No (30/25 s duration)	-
Sil et al. [69]	Full wave rect.	No (30 s duration)	-
Fricke et al. [70]	High-pass (20 Hz), low-pass (400 Hz), full-wave rect.	Not declared	ACC data used to segment sEMG data around gait cycles

**Table 12:** Feature extraction methods, feature selection algorithms and best sEMG features for the disease differential diagnosis task.

Study	Feature Extraction	Feature Selection	Best sEMG Features
Tang et al. [67]	Automatic + Cross-attention	✗	-
Xing et al. [68]	Handcrafted	✓ (Importance)	Dom_freq_ext, Dom_freq_fle, Ave_amp_ext, Ave_amp_fle
Sil et al. [69]	Handcrafted	✓ (RFECV + SHAP)	Avg_pow_ext_left_rest, pow_ext_left_rest, freq_ext_left_pos+wt
Fricke et al. [70]	Handcrafted + Automatic (CNN)	✓ (PCA)	IEMG, SSI, VAR, RMS, AOP, SM1, PKF

✓: used a feature selection technique; ✗: did not use a feature selection technique; Dom_fre_ext: dominant frequency of extensors; Dom_fre_fle: dominant frequency of flexors; Ave_amp_ext: average amplitude of extensors; Ave_amp_fle: average amplitude of flexors; IEMG: integrated EMG; SSI: simple square integral; VAR: variance; RMS: root mean square; AOP: area of power; SM1: spectral moment; PKF: peak frequency.

**Table 13:** Dataset splitting, training method, models evaluated, and performance reached by each study involved in differential diagnosis using sEMG sensors. In the “Models” column, the architecture written in italic is indicative of the best model reaching the performance reported in the “Performance” column. Fricke et al. [70] performance reported only for the three-class classification problem.

Study	Train, Test, Val Split (%)	Training Method	Models	Hyperparameters	Performance
Tang et al. [67]	70, 30, (20)	CV	*CNN*	Not declared	ACC = 97.18%
Xing et al. [68]	80, -, 20	10-fold CV	RF, *XGB*, LR, SVM, Ridge, BP, CNN	Grid search	ACC = 85%
Sil et al. [69]	80, -, 20	Nested CV (outer k = 10 inner k = 5)	RF, ETR, *XGB*, LGBM, SVM, ANN, LR	Bayesian optimization	ACC = 91%
Fricke et al. [70]	60, 2, 38	LOSO	*CNN*, SVM, kNN	Grid search	ACC = 83.8%

XGB: extreme gradient boosting; ETR: extra tree regressor; LGBM: light gradient boosting machine.

**Table 14:** Dataset and acquisition protocol information for the FoG detection in PD task using EMG data. All papers used the same publicly available dataset ([71,95]), but their choices differ between number of patients, sensors considered and muscles observed. The protocol adopted, however, remains the same.

Study	Subjects	Sensors	Muscles	Protocol
Zhang et al. [71]	12 PD (6 male, 6 female)	MOVE system (EEG + sEMG) TDK MPU6050 (ACC + SC)	GS right leg, TA both legs	TUG test with “FoG-triggering” elements
Murtaza et al. [72]	12 PD	sEMG, EEG, ACC, SC	GS right leg, TA both legs	"
Gupta et al. [73]	10 PD (more FoG)	sEMG, ACC, EEG	GS right leg, TA both legs	"
Munjal et al. [74]	12 PD	sEMG, ACC, SC	TA both legs	"
Hou et al. [75]	12 PD	ACC, sEMG, 4 EEG channels	TA both legs	"

EEG: electroencephalogram; SC: skin conductance; GS: gastrocnemius; TA: tibialis anterioris; TUG: timed up and go.

**Table 15:** Preprocessing and windowing choices for the studies that implemented FoG detection and early prediction using sEMG signals. In the last column, additional techniques that might have been used alongside the typical preprocessing steps are reported.

Study	Preprocessing	Windowing	Other Techniques
Zhang et al. [71]	Resampling (500 Hz), bandpass (10–500 Hz), notch (50 Hz), normalization	3 s length 0.3 s sliding step, PFG-based labeling (th = 80%)	Cubic interpolation for ACC calibration
Murtaza et al. [72]	Resampling (500 Hz)	3 s length, 90% overlap, PFG-based labeling (different thresholds)	Testing of different windows
Gupta et al. [73]	Bandpass (10–450 Hz), downsampling (500 Hz)	0.128 s length before FoG event, labeled as FoG	-
Munjal et al. [74]	Same as Zhang et al. [71]	3 s length, 10% overlap, majority-voting labeling	Interpolation
Hou et al. [75]	Not declared	3 s length	-

**Table 16:** Feature extraction methods and choices for the FoG detection or early prediction task using sEMG data for Parkinson’s disease patients.

Study	Feature Extraction	Feature Selection	Best sEMG Features
Zhang et al. [71]	Handcrafted	✗	MAV, ZC, SSC, WL
Murtaza et al. [72]	Handcrafted	✗	MAV, ZC, SSC, WL
Gupta et al. [73]	Handcrafted + Automatic	✗	-
Munjal et al. [74]	Handcrafted	✗	MAV, ZC, SSC, WL
Hou et al. [75]	Automatic	✗	-

✓: used a feature selection technique; ✗: did not use a feature selection technique; MAV: mean absolute value; ZC: zero crossings; SSC: slope sign change; WL: waveform length.

**Table 17:** Details regarding dataset split percentages, training method, models compared for FoG detection, hyperparameter optimization and selection and performance metrics highlighting the model’s achievements. The italic model in the “Models” column represents the one selected as the best-performing for this task.

Study	Train, Test, Val Split (%)	Training Method	Models	Hyperparameters	Performance
Zhang et al. [71]	80, 20, -	CV	*SVM with* *RBF kernel*	Grid search	Acc ≈ 96%
Murtaza et al. [72]	80, 20, -	10 reps	*SVM*, RF, XGB	Grid search	Acc = 98.86%
Gupta et al. [73]	Not declared	10-fold CV, LOSO	SVM, LDA, DT, RF, NN, *1D CNN–LSTM*	Reported	Acc = 94.20%
Munjal et al. [74]	90, -, 10	5-fold CV	*DNN*	Reported	Acc = 89%
Hou et al. [75]	Not declared	LOSO	*DNN*	Reported	Acc = 85%

SVM: support vector machine; RBF: radial basis function; RF: random forest; XGB: extreme gradient boosting; LDA: linear discriminant analysis; DT: decision tree; NN: neural network; CNN: convolutional neural network; LSTM: long short-term memory; DNN: deep neural network.

**Table 18:** Overview of the datasets, sensors, recorded muscles, and acquisition protocols adopted in studies focusing on tremor analysis in Parkinson’s disease using sEMG signals.

Study	Subjects	Sensors	Muscles	Protocol
Wang et al. [76]	39 PD (H&Y = 2.5, 3)	Nicolet (sEMG)	EDS, FDS, TA, GS	Relaxed against gravity, DBS off/on
Davoudi et al. [77]	10 PD (UPDRS = 3)	Gtech (sEMG)	Wrist flexor	Sitting during auditory biofeedback
Zanini et al. [78]	5 (4 PD, 1 ET)	Delsys Trigno (sEMG)	Wrist flexor and extensor	Arms extended: isometric, grab, pinch
Lin et al. [79]	37 (24 PD UPDRS = 0–2, 13 HC)	Shimmer3 (inertial + sEMG)	ECU, FCU right arm	Hold hands stationary + lift
Qin et al. [80]	147 PD (UPDRS = 0–4)	Not declared (sEMG)	Biceps	Not declared
Farhani et al. [81]	17 PD	Myo armband (sEMG)	FCU, ECU, FDS, EDS, FCR, ECR	Resting, postural, grabbing, writing

EDS: extensor digitorum superficialis; FDS: flexor digitorum superficialis; TA: tibialis anterioris; GS: gastrocnemius; ECU: extensor carpi ulnaris; FCU: flexor carpi ulnaris; FCR: flexor carpi radialis; ECR: extensor carpi radialis.

**Table 19:** Summary of the signal processing pipelines adopted in tremor-related studies, including preprocessing methods, windowing configurations, and additional techniques applied to sEMG signals.

Study	Preprocessing	Windowing	Other Techniques
Wang et al. [76]	Bandpass (20–200 Hz), full-wave rect., envelope extraction	1 s length	Non-RT-related signals removal
Davoudi et al. [77]	Bandpass (10–100 Hz), MVC norm.	3 s length	Unification of the 2 EMG channels
Zanini et al. [78]	Low-pass (500 Hz), notch (50 Hz), rect., smooth., norm.	0.2 s length (predict) based on previous 2 s	Comparison with smoothed version of raw scaled from −1 to 1
Lin et al. [79]	High-pass (0.25 Hz)	3 s length	Average value correction for outlier removal
Qin et al. [80]	Not declared	2.048 s length	-
Farhani et al. [81]	Low-pass (20 Hz)	250 ms length, 50% overlap	-

**Table 20:** Overview of feature extraction methods, selection techniques, and relevant sEMG features used in sEMG-based tremor analysis studies.

Study	Feature Extraction	Feature Selection	Best sEMG Features
Wang et al. [76]	Handcrafted	✓ (Paired T-tests, Spearman corr.)	wRMS, peak(f)PSD, MNP
Davoudi et al. [77]	Not applicable	-	-
Zanini et al. [78]	Automatic	✗	-
Lin et al. [79]	Automatic (FCN, LSTM)	✓ (Attention mechanism)	-
Qin et al. [80]	Handcrafted	✓ (Statistical importance)	ZC, MDF, VAR, MSF, RMS, WL, LOG
Farhani et al. [81]	Automatic	✗	-

✓: used a feature selection technique; ✗: did not use a feature selection technique; wRMS: weighted root mean square; peak(f)PSD: peak frequency power; MNP: mean amplitude power; ZC: zero crossing; MDF: median frequency; VAR: variance; MSF: mean spectral frequency; RMS: root mean square; WL: waveform length; LOG: log detector.

**Table 21:** Overview of data splitting strategies, training methodologies, adopted models, hyperparameter tuning approaches, and performance metrics reported in studies focusing on tremor analysis in Parkinson’s disease.

Study	Train, Test, Val Split (%)	Training Method	Models	Hyperparameters	Performance
Wang et al. [76]	Not declared	LOSO	*GPR*	Not declared	r = 0.47, p = 0.003
Davoudi et al. [77]	Not applicable	-	*AdaBoost* *clustering*	Not declared	-
Zanini et al. [78]	80, 20, (10)	-	MLP, LSTM, MLP + LSTM, *MLP autoenc.*, LSTM autoenc.	Reported	γ = 0.01
Lin et al. [79]	75, 25, -	-	SVM, FCN, LSTM, LSTM + FCN, *LSTM + FCN + Att.*	Reported	Acc = 91.78%
Qin et al. [80]	80, 20, -	Similarity learning	SVM, MLP, kNN, Bayes, SNet_mod, *SNet*	Reported	Acc = 90.55%
Farhani et al. [81]	80, 20, -	LOSO, warm initialization	*BiLSTM*	NAS (aging)	Acc ≈ 89%

LOSO: leave-one-subject-out; GPR: Gaussian process regression; MLP: multilayer perceptron; LSTM: long short-term memory; SVM: support vector machine; FCN: fully connected network; Att.: attention mechanism; kNN: k-nearest neighbor; BiLSTM: bidirection long short-term memory; NAS: neural architecture search.

**Table 22:** Summary of dataset size, sensors used during the acquisition, muscles observed and acquisition protocol followed for each study aiming at characterizing a symptom PD or patients’ gait.

Study	Subjects	Sensors	Muscles	Protocol
Lin et al. [82]	53 (13 HC, 40 PD, UPDRS = 0–3)	Shimmer3 (sEMG + IMU)	ECU, FCU	PSMH, FT, HM
Kozulin et al. [83]	15 (7 HC, 8 PD, H&Y = 2–3)	Delsys Trigno (sEMG)	FCR, ED	FT, HM
Guo et al. [84]	41 (12 HC, 29 PD)	Not declared (sEMG + IMU + FP)	TA, GS	Sitting + walking
Alves et al. [85]	10 (5 HC, 5 PD)	Developed (sEMG)	Wrist flexor and extensor	Active movements + serious game
Liu et al. [86]	21 (10 HC, 11 PD UPDRS = 1–2)	Noraxon’s Ultium (sEMG)	TA, GS	Walking straight
Haufe et al. [87]	6 PD (H&Y = 1–3)	Opal APDM, FREEMG BTS (IMU + sEMG)	TA, S, GM, GL, VL	Walking ellipsoidal path
Bengacemi et al. (2021) [88]	17 (9 HC, 8 PD)	Delsys Trigno (sEMG)	RightS	Walking straight
Bengacemi et al. (2024) [89]	17 (9 HC, 8 PD)	Delsys Trigno (sEMG)	RightS	Walking straight

ECU: extensor carpi ulnaris; FCU: flexor carpi ulnaris; PSMH: pro/supination movements of hands; FT: finger tapping; HM: hand movements; FP: foot pressure; TA: tibialis anterioris; GS: gastrocnemius; S: soleus; GM: gastrocnemius medialis; GL: gastrocnemius lateralis; VL: vastus lateralis; RigthS: right soleus.

**Table 23:** Overview of the signal preprocessing procedures, windowing strategies, and additional processing techniques adopted in studies focusing on gait analysis and motor symptom assessment in Parkinson’s disease using sEMG signals.

Study	Preprocessing	Windowing	Other Techniques
Lin et al. [82]	Not declared	Not declared	LSTM–VAE-based method for data balancing
Kozulin et al. [83]	Remove DC offset, bandpass (10–100), rect., envelope	1 s length 500 ms overlap (Hanning)	-
Guo et al. [84]	Not declared	5 s length	-
Alves et al. [85]	High-pass (20 Hz), low-pass (500 Hz), notch (60 Hz), MVC norm., env.	Not declared	EMD + PSD analysis
Liu et al. [86]	Bandpass (20–450 Hz), norm., env.	Gait cycles	Wavelet analysis on segmented sEMG
Haufe et al. [87]	Bandpass, rect., downsample (250 Hz), smoothness, norm.	1 s length	-
Bengacemi et al. (2021) [88]	Not declared	66.45 ms overlapping	Test of optimal window duration
Bengacemi et al. (2024) [89]	Not declared	80 ms overlapping	Test of optimal window duration

LSTM: long short-term memory; VAE: variational autoencoder; EMD: empirical mode decomposition; PSD: power spectral density.

**Table 24:** Summary of feature engineering strategies adopted in studies addressing gait analysis and motor symptom assessment, including feature extraction techniques, selection methods, and the most relevant sEMG features.

Study	Feature Extraction	Feature Selection	Best sEMG Features
Lin et al. [82]	Automatic + Handcrafted	✓ (ResNet for feature shrinking)	MI, KLdiv, CCcoeff, SampEn, PermEn
Kozulin et al. [83]	Handcrafted	✗	(1) NP, MDA, AvgTime, (2) MAV1, MAV2, AppEn, SampEn
Guo et al. [84]	Handcrafted	✓ (with single gait disorder models)	IEMG, AEMG, RMS
Alves et al. [85]	Not applicable	-	-
Liu et al. [86]	Automatic	✗	-
Haufe et al. [87]	Handcrafted	✗	21 sEMG features
Bengacemi et al. (2021) [88]	Handcrafted	✓ (Performance comparison)	LWE
Bengacemi et al. (2024) [89]	Handcrafted	✓ (Performance comparison)	LWE

✓: used a feature selection technique; ✗: did not use a feature selection technique; MI: mutual information; KLdiv: Kullback–Leibler divergence; CCcoeff: cross-correlation coefficient; SampEn: sample entropy; PermEn: permutation entropy; NP: number of peaks; MDA: median amplitude; AvgTime: average time interval between found peaks; MAV1, MAV2: modifications of mean absolute value; AppEn: approximate entropy; IEMG: EMG integral value; AEMG: mean EMG; RMS: root mean square; DWT: discrete wavelet transform; LWE: log wavelet energy.

**Table 25:** Overview of data splitting strategies, training methodologies, adopted models, hyperparameter tuning approaches or hyperparameters chosen for model definition, and performance metrics reported in studies focusing on gait analysis and motor symptom assessment in Parkinson’s disease.

Study	Train, Test, Val Split (%)	Training Method	Models	Hyperparameters	Performance
Lin et al. [82]	75, 25, -	4-fold CV	*LSTM* *+ FCN*	Grid search	Acc > 85%
Kozulin et al. [83]	-	LOSO (ON, OFF, HC each test)	*RF*	Not declared	r = 0.17 (HM), = 0.63 (FT)
Guo et al. [84]	Not declared	Not declared	*RF*, SVM, KNN	Not declared	Acc = 85.7%
Alves et al. [85]	Not applicable	-	Spearman corr. + ANOVA	-	r > 0.7
Liu et al. [86]	-	LOSO (2 HC, PD1, PD2 each test)	*Fusion of* *conv.* *nets*	Not declared	Acc = 92.86%
Haufe et al. [87]	-	LOSO	*Multiple lin.* *regression*	Not declared	F1 ≈ 89%
Bengacemi et al. (2021) [88]	Not declared	Not declared	*HMM*	Baum- Walsh alg.	Acc = 100%
Bengacemi et al. (2024) [89]	53, 47, -	Not declared	*HMM*	Not declared	Acc = 93.37%

LOSO: leave-one-subject-out; ON/OFF: on or off medication state; PD1/PD2: patients with severity 1, 2; LSTM: long short-term memory; FCN: fully connected network; RF: random forest; SVM: support vector machine; kNN: k-nearest neighbor; HMM: hidden Markov model; HM: hand movement; FT: finger tapping.

**Table 26:** Dataset size variability per task measured as mean ± standard deviation in terms of number of subjects independently of whether they are PD, HC, or other classes. The last row considers all studies regardless of the assigned category.

Category	Dataset Size Variability (Number of Subjects)
Diagnosis	27.75±22.60
Assessment	29.33±15.04
Differential Diagnosis	242.75±183.01
FoG Prediction	11.60±0.89
Tremor Quantification	42.50±53.06
Symptom Quantification	28±18.28
Gait Analysis	13.33±6.35
All	55.43±97.14

**Table 27:** Availability of the datasets used in the reviewed studies, grouped by research topic. The table reports whether the datasets employed in each work are publicly available, available upon request, or private. Symbols indicate the level of accessibility.

Topic	Study	Dataset Availability
Diagnosis and Assessment	Loconsole et al. [61]	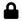
	Rezaee et al. [62]	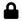
	Saikia et al. [63]	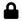
	Adem et al. [64]	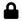
	Kleinholdermann et al. [65]	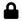
	Sun et al. [66]	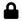
Diff. Diagnosis	Tang et al. [67]	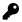
	Xing et al. [68]	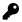
	Sil et al. [69]	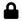
	Fricke et al. [70]	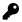
FoG	Zhang et al. [71]	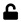 [95]
Tremor	Wang et al. [76]	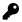
	Davoudi et al. [77]	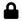
	Zanini et al. [78]	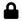
	Lin et al. [79]	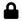
	Qin et al. [80]	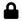
	Farhani et al. [81]	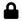
Gait Analysis and Motor Symptoms	Lin et al. [82]	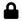
	Kozulin et al. [83]	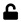 [101]
	Guo et al. [84]	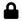
	Liu et al. [86]	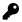
	Haufe et al. [87]	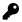
	Bengacemi et al. (2021,2024) [88,89]	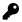 [102]
	D’Amico et al. [100]	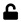 [100]

LEGEND 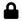: private dataset; 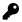: dataset available upon request; 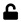: publicly available dataset.

**Table 28:** Preprocessing steps, related techniques, and purpose employed by the reviewed studies for sEMG signal analysis. The seven studies that did not provide descriptions of their preprocessing procedures were excluded from this assessment.

Preprocessing Step	Technique	Studies	Purpose
Filtering	Bandpass (≈10–500 Hz)/ low-pass (≈500 Hz) and high-pass (≈10 Hz), notch (50–60 Hz)	19	Noise removal
Resampling	Upsampling or downsampling	5	Uniform signal to other modalities
Rectification	Full-wave rectification	6	Amplitude envelope
Smoothing	RMS/moving average	3	Signal cleaning
Envelope extraction	Low-pass filtering, RMS, moving average, or Hilbert transform	4	sEMG amplitude envelope over time
Normalization	%MVC, Z-score, min–max	10	Reduce inter-subject variability
Segmentation	Sliding window	19	Feature extraction

**Table 29:** Typical domains from which features are manually extracted from sEMG signals collected from Parkinson’s patients. The column “Studies” indicates the number of reviewed papers that extracted features from the corresponding domain.

Feature Type	Common Features	Studies	Meaning
Time domain	RMS, MAV, WL, ZC	17	Muscle activation amplitude
Frequency domain	MNF, MDF	8	Muscle fatigue/ tremor frequency
Power domain	PKF, MNP	4	Muscle activation power and oscillatory activity
Time–frequency	Wavelet coefficients	2	Non-stationary and non-linear patterns
Non-linear	Entropy, skewness	3	Signal complexity

**Table 30:** Validation strategies adopted in the reviewed studies.

Validation Technique	Studies
Train–test split	4
4-fold CV	1
5-fold CV	4
10-fold CV	5
LOSO	7
Not declared	9

**Table 31:** Comparison between machine learning and deep learning approaches’ performance in sEMG-based Parkinson’s disease analysis. For each study, the best-performing model is reported in brackets under the “Approach” column. Performance is expressed as classification accuracy, which is the only metric consistently reported across the included studies; papers not reporting accuracy were excluded from this analysis.

Paper	Approach	Accuracy (%)
Loconsole et al. [61]	DL (ANN)	97.84
Rezaee et al. [62]	ML (SVM)	99.42
Adem et al. (diagnosis) [64]	ML (SVM)	94.15
Adem et al. (assessment) [64]	ML (SVM)	93.33
Sun et al. [66]	ML (DT)	96.00
Tang et al. [67]	DL (CNN)	97.18
Xing et al. [68]	ML (XGB)	85.00
Sil et al. [69]	ML (XGB)	91.00
Fricke et al. [70]	DL (CNN)	83.80
Zhang et al. [71]	ML (SVM)	96.00
Murtaza et al. [72]	ML (SVM)	98.86
Gupta et al. [73]	DL (CNN–LSTM)	94.20
Munjal et al. [74]	DL	89.00
Hou et al. [75]	DL	85.00
Lin et al. [79]	DL (LSTM–FCN)	91.78
Qin et al. [80]	DL (CNN)	90.55
Farhani et al. [81]	DL (LSTM)	89.00
Lin et al. [82]	DL (LSTM–FCN)	85.00
Guo et al. [84]	ML (RF)	85.70
Liu et al. [86]	DL (CNN)	92.86
Bengacemi et al. (2021) [88]	ML (HMM)	100
Bengacemi et al. (2024) [89]	ML (HMM)	93.37

**Table 32:** Proposed checklist of minimum requirements for future potential sEMG-based Parkinson’s disease studies to improve reproducibility and comparability.

Category	Parameter	Recommended/Common Practice
Acquisition protocol	Sensor type	sEMG and multimodality (e.g., IMU, EEG, SC)
	Sampling frequency	Typically $f_{s}\geq$ 1000 Hz
	Monitored muscles	- TA, GM for lower limbs - FCU, ECU, FCR, ECR for upper limbs
	Electrode placement	Follow SENIAM guidelines
	Experimental protocol	Clinical tests (following MDS-UPDRS)
	Recording conditions	- Laboratory for clinical validation - Home environment for real-world investigation
Dataset	Number of subjects	>50 subjects per category
	Class distribution	Balanced both in type of subject and severity
	Disease severity	- UPDRS for symptoms severity - H&Y for general disease evaluation
	Data availability	Publicly available or available upon request
Signal Processing	Filtering	- Band-pass 20–450 Hz - Notch 50/60 Hz
	Envelope extraction	Rectification, smoothing, low-pass filtering (5 Hz)
	Normalization	%MVC if available, Z-score, Min-Max
	Segmentation	- Window length between 250 ms and 3 s - Overlap between 30–90%
Feature Engineering	Method	- Manual extraction for ML models - Automatic extraction for DL models
	Feature types	Time, frequency, time-frequency, non-linear
	Features	RMS, MAV, WL, ZC, entropy, MNF, MDF
	Feature selection	PCA, importance analysis, ensemble methods
Model Evaluation	Model type	ML (SVM, RF) or DL (CNN, LSTM)
	Hyperparameters	Gridsearch, Bayesian optimization, NAS
	Training and validation	LOSO
	Performance metrics	Accuracy, F1-score

## Data Availability

The original contributions presented in this study are included in the article. Further inquiries can be directed to the corresponding author(s).

## Abbreviations

The following abbreviations are the most frequently used in this manuscript; additional abbreviations are defined at their first occurrence or within the corresponding tables.

PD	Parkinson’s disease
MDS-UPDRS	Movement Disorder Society Unified Parkinson’s Disease Rating Scale
sEMG	surface electromyography
IMU	inertial measurement unit
ML	machine learning
DL	deep learning
AI	artificial intelligence
H&Y	Hoehn and Yahr scale
FoG	freezing of gait
IoT	Internet of Things
ECG	electrocardiogram
EEG	electroencephalogram
ET	essential tremor
SENIAM	Surface EMG for Non-Invasive Assessment of Muscles
RMS	root mean square
MAV	mean absolute value
ZC	zero crossings
PSD	power spectral density
MNF	mean frequency
MDF	median frequency
%REC	recurrence
%DET	determinism
CD	correlation dimension
sampEn	sample entropy
HC	healthy control
SVM	support vector machine
kNN	k-nearest neighbor
DT	decision tree
LDA	linear discriminant analysis
ANN	artificial neural network
DNN	deep neural network
FCNN	fully connected neural network
CNN	convolutional neural network
RNN	recurrent neural network
LSTM	long short-term memory
MVC%	maximum voluntary contraction
PCA	principal component analysis
WL	waveform length
LOSO	leave-one-subject-out
CV	cross-validation
CWT	continuous wavelet transform
SC	skin conductance
TUG	timed up and go
PFG	percentage of FoG points
RT	resting tremor
DWT	discrete wavelet transform
DBS	deep brain stimulation
FES	functional electrical stimulation
